# LncRNA MEG3 exacerbates diabetic cardiomyopathy via activating pyroptosis signaling pathway

**DOI:** 10.3389/fphar.2025.1538059

**Published:** 2025-04-02

**Authors:** Shengnan Zhuo, Yifeng Liu, Siyuan Wang, Zhuoling Chen, Xuran Shi, Yangjunna Zhang, Dengfeng Xu, Jingjin Hu, Yin Wang, Xuefeng Qu

**Affiliations:** ^1^ School of Pharmacy, School of Food Science and Engineering, Hangzhou Medical College, Hangzhou, Zhejiang, China; ^2^ Department of Health Monitoring, Bazhong Center for Disease Control and Prevention, Bazhong, Sichuan, China; ^3^ School of Public Health, Hangzhou Medical College, Hangzhou, Zhejiang, China

**Keywords:** diabetic cardiomyopathy, *lncMEG3*, miR-223, pyroptosis, cardiac function

## Abstract

**Background:** Diabetic cardiomyopathy (DCM) is a prevalent complication observed in diabetic patients. The long non-coding RNA maternally expressed gene 3 (*lncMEG3*) has been found to be intricately associated with myocardial infarction and heart failure. However, the role of *lncMEG3* in DCM remains unclear. The present study was designed to investigate the role of *lncMEG3* in DCM and elucidate the underlying molecular mechanisms.

**Methods:** The diabetic mouse model was established through intraperitoneal injection streptozotocin (STZ). The heart-targeted adeno-associated virus carrying *lncMEG3* interfering RNA (AAV9-shMEG3) was administered via tail-vein injection to induce silencing of *lncMEG3* in diabetic mice. Echocardiography was performed to evaluate cardiac function, while hematoxylin and eosin (H&E) staining and Masson trichrome staining were employed for the detection of cardiac remodeling. The underlying mechanisms were investigated using Western blot and real-time PCR (qPCR).

**Results:** The expression of *lncMEG3* was increased in hearts with DCM and in AC16 cardiomyocytes treated with high glucose. The knockout of *lncMEG3* reduced inflammation, cardiac fibrosis and myocardial hypertrophy, and improved cardiac dysfunction in diabetic mice. In diabetic mice, the activation of the nucleotide-binding oligomerization domain-like receptor pyrin domain containing 3 (NLRP3)-inflammasome was observed, whereas silencing of *lncMEG3* resulted in a reduction in NLRP3 inflammasome activation. Mechanistically, we discovered that *lncMEG3* specifically functions as a competitive inhibitor of *miR-223*. Moreover, the use of *miR-223* antisense oligonucleotide (AMO) counteracted the suppressive effects of *lncMEG3* knockdown on NLRP3 inflammasome activation induced by high glucose *in vitro*.

**Conclusion:** *LncMEG3* exacerbates DCM by enhancing NLRP3 inflammasome activation through attenuating *miR-223*-mediated degradation of NLRP3 in the hearts of individuals with diabetes.

## Graphical Abstract

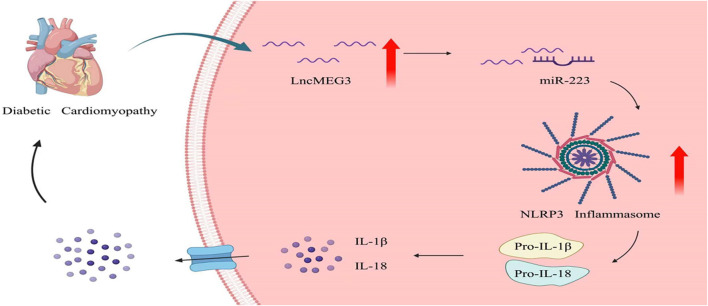

## 1 Introduction

Diabetes is a prominent global public health issue, contributing to approximately one in nine deaths among individuals aged 20–79 years (Saeedi et al., 2020). The pathogenesis of diabetes primarily involves hyperglycemia, which triggers a multitude of metabolic signaling pathways, leading to the induction of oxidative stress and inflammation, ultimately resulting in the development of various complications. Diabetic cardiomyopathy (DCM) is a prominent complication observed in patients with diabetes mellitus and represents a significant contributor to mortality (Dillmann, 2019; Nakamura et al., 2022). The pathogenesis of DCM involves inflammation, mitochondrial dysfunction, apoptosis and oxidative stress, which ultimately lead to myocardial hypertrophy, interstital fibrosis of the myocardium, cardiac dysfunction (Zhao et al., 2022). Among them, inflammation stands out as one of the predominant pathogenic mechanisms in DCM. The activation of nucleotide-binding oligomerization domain-like receptor family pyrin domain containing 3 (NLRP3) inflammasome-mediated pyroptosis has been demonstrated to induce the release of proinflammatory cytokines into cardiac myocytes, thereby promoting collagen deposition and contributing to the development of left ventricular (LV) hypertrophy (Yan et al., 2022). The therapeutic intervention of NLRP3 gene silencing demonstrated significant improvements in cardiac hypertrophy, pyroptosis, fibrosis, and cardiac function (Luo et al., 2014).

Long non-coding RNA (LncRNA) refers to a class of RNA transcripts longer than 200 nucleotides that lack protein-coding capacity (Bridges et al., 2021), yet play a crucial regulatory role in cardiovascular diseases, as supported by substantial evidence (Holdt and Teupser, 2012; Qu et al., 2017; Viereck et al., 2016). Maternally expressed gene 3 (*MEG3*) is the first lncRNA identified to possess tumor suppressor activity. It exhibits abundant expression in cardiac tissue and has been demonstrated to be upregulated in heart diseases, including myocardial hypertrophy, myocardial fibrosis, and myocardial infarction (Li et al., 2019; Zhang et al., 2019). Recently, Piccoli et al. (2017) reported that *lncMEG3* plays a crucial role in promoting cardiac fibrosis, whereas knockout of *lncMEG3* effectively prevents the development of cardiac fibrosis and diastolic dysfunction. Moreover, attenuation of *lncMEG3* provides myocardial cell protection against ischemia reperfusion-induced apoptosis through modulation of the *miR-7*-5p/PARP1 pathway (Zou et al., 2019). However, the relationship between DCM and *lncMEG3* remains unclear.

In recent years, *lncMEG3* has emerged as a crucial regulator of gene expression in cardiovascular diseases, functioning as a competitive endogenous RNA (ceRNA) that binds microRNAs (miRNAs). The expression of *miR-223* is downregulated in endothelial cells induced by high glucose and high fat, while overexpression of *miR-223* suppresses the activation of NLRP3 inflammasome (Deng et al., 2020). In the presence of elevated blood glucose levels, mitochondria excessively generate reactive oxygen species (ROS), which are recognized by thioredoxin-interacting protein (TXNIP). TXNIP directly interacts with NLRP3, leading to inflammasome activation and subsequent cleavage of pro-Caspase-1 into active Caspase-1 (Cas-1 cut). Cas-1 cut leads to the cleavage of downstream cytokines pro-Interleukin-1 beta (pro-IL-1β) and pro-Interleukin-18 (pro-IL-18) into their mature forms IL-1β, IL-18, thereby inducing cardiomyocyte damage and subsequently resulting in myocardial hypertrophy, fibrosis, and impaired cardiac function (Denes et al., 2012; Vande Walle and Lamkanfi, 2020). Several studies have shown that NLRP3 inflammasome is involved in the development of DCM (Liu et al., 2022; Zhang et al., 2022).

In this study, we have demonstrated that the expression of *lncMEG3* is upregulated in the hearts of mice with DCM and in AC16 cardiomyocytes induced by high glucose (HG). Knockdown of *lncMEG3* attenuates inflammation and NLRP3 inflammasome-mediated pyroptosis, thereby ameliorating myocardial hypertrophy, cardiac fibrosis, and cardiac dsyfunction through modulation of the *miR-223*/NLRP3 pathway. Therefore, elucidating the precise role of *lncMEG3* in DCM and unraveling its intricate molecular regulatory network could potentially provide novel targets for the development of effective functional foods and innovative pharmaceutical interventions.

## 2 Methods

### 2.1 Animals

Eight-week-old male C57BL/6 mice were provided by the Hangzhou Medical College. The mice were housed under standard conditions, with a temperature maintained at 22°C ± 1°C, humidity at 55% ± 5%, and a light-dark cycle of 12 h each. The mice were provided *ad libitum* access to food and water until sacrifice. All experimental procedures adhered to the guidelines approved by the Ethics Committees of Hangzhou Medical College (2018-045).

### 2.2 Mouse model of diabetic cardiomyopathy

Sixty mice were deprived of food but provided with water normally for 4 h prior to streptozotocin (STZ, Sigma-Aldrich, United States, V900890) treatment, from 7 a.m. to 11 a.m. At 11 a.m., the mice received intraperitoneal injections of STZ at a dose of 50 mg/kg body weight per day, which dissolved in a 10 mmol/L citrate buffer, for five consecutive days to induce diabetes. The control (Ctl) mice were treated with an equivalent volume of citrate buffer only. One week later, the animals underwent a 4-hour fasting period, during which the treatment group’s fasting blood glucose concentrations were measured using a glucometer (LifeScan, United States). Animals with fasting blood glucose concentration higher than 16.7 mmol/L were considered as successful establishment of diabetes model. The mice were maintained for a duration of 12 weeks following STZ injection with recording the fasting blood glucose, body weight, and cardiac function.

### 2.3 Lentiviral *lncMEG3* shRNA vector building

The *lncMEG3* short hairpin RNA (shRNA) was inserted into the heart-targeted adeno-associated virus 9 vector (cTNTp-EGFP-MIR155(MCS)-WPRE-SV40 PolyA, Genechem, China) (Chen et al., 2024). The AAV9 vector contained cTnT (cardiac-specific troponin T) promoter. And AAV9-cTnT-GFP was served as negative control. The virus was suspended in 0.9% NaCl solution with a viral titer of 1E+12 vg/mL, and subsequently, 200 μL of AAV9-shMEG3 were administered via tail vein injection to diabetic mice. Cardiac function and pathological changes were evaluated 12 weeks post-diabetes induction. The mouse *lncMEG3* shRNA sequences are: 5′- ATT​CCA​GAT​GAT​GGC​TTT​GGC​GTT​TTG​GCC​ACT​GAC​TGA​CGC​CAA​AGC​TCA​TCT​GGA​AT-3′.

### 2.4 Echocardiography

VINNO 6 Imaging System (VINNO, China) was employed for the assessment of cardiac function. The M-mode images of the left ventricle (LV) were recorded over three consecutive cardiac cycles. These images were used to calculate LV end-diastolic septal thickness (IVSd), LV end-systolic septal thickness (IVSs), LV end-diastole internal dimension (LVIDd), LV end-systolic internal dimension (LVIDs), LV end-diastole posterior wall thickness (LVPWd), LV end-systolic posterior wall thickness (LVPWs), ejection fraction (EF), and shortening fraction (FS).

### 2.5 Hematoxylin and eosin (H&E) staining

The heart tissues were freshly collected, fixed in 4% paraformaldehyde (Biosharp, China) for 24 h at room temperature. After dehydration, the hearts were embedded with paraffin, and sectioned into 5 μm thin slices. Sections were imaged at ×400 magnification using light microscopy (CKX53, Olympus, Japan). Heart architectures were observed for myocardial hypertrophy in transverse heart sections. Utilizing ImageJ software (NIH, United States), quantification of 5 images per section was performed to measure the cardiomyocyte area (μm^2^) and determine myocardial hypertrophy.

### 2.6 Masson’s trichrome staining

The heart tissues were freshly collected, fixed in 4% paraformaldehyde (Biosharp, China) for 24 h at room temperature. After dehydration, the hearts were embedded with paraffin, and sectioned into 5 μm thin slices. The heart sections stained with a Masson’s trichrome were examined using a microscope (CKX53, Olympus, Japan) at ×200 magnification, and the collagen deposition of myocardial interstitium and perivascular was identified as blue staining, which is indicative of the presence of fibrotic tissue. The myocardial fibrotic area was analyzed using Image-Pro Plus 5.0 software.

### 2.7 Culture and treatment of human cardiomyocyte line AC16 cardiomyocytes

The human cardiomyocyte line AC16 cardiomyocytes were purchased from Otwo biotechnology company Limited in Shenzhen, China. The AC16 cardiomyocytes were cultured in DMEM (HyClone, United States) medium supplemented with 5.5 mmol/L glucose and 10% bovine serum (BI, Israel), and maintained at a temperature of 37°C in a humidified atmosphere containing 5% CO_2_. Following cell transfection, the AC16 cardiomyocytes were exposed to either a normal glucose medium (5.5 mmol/L) or a high glucose (HG) medium (35 mmol/L) for 24 h prior to subsequent experimental procedures.

### 2.8 Transfection of shRNA and AMO-223 to cells

*LncMEG3* shRNA and *miR-223* inhibitor (AMO-223) were designed and synthesized by GenePharma (GenePharma, China). The transfection of *lncMEG3* shRNA and AMO-223 into AC16 cardiomyocytes was conducted using Lipofectamine 2000 transfection reagent (Invirogen, United States). The shRNA and AMO-223 were diluted in Opti-MEM medium and transfected into the cells. Following a 6-hour transfection period, the DMEM medium was replaced, and cell culture was continued for an additional 48 h.

### 2.9 Cellular immunofluorescence

The AC16 cardiomyocytes were seeded at a density of 1.0 × 10^5^ cells per well in a 12-well plate to ensure even distribution, followed by cell transfection, exposure to high glucose, and other treatments according to the experimental groups. Subsequently, the cells were fixed using 4% paraformaldehyde (Beyotime, China) for 15 min at room temperature. After that, 1.5 mL of 0.5% Triton X-100 (Beyotime, China) was added to each well for permeabilization and incubated for 15 min. Subsequently, the fixed cells were blocked with 5% bovine serum albumin (BSA, Beyotime, China) at room temperature for a duration of 1 h, followed by overnight incubation on a shaker at 4°C with diluted α-actin antibody (Abcam, ab137346, United Kingdom). The next day, incubate the Alexa Fluor^®^ 594 fluorescent secondary antibody (abcam, ab150080, United Kingdom) at room temperature for 2 h and then add Hoechst 33258 (Beyotime, China) diluted with PBS to a final concentration of 5 μg/mL for 5 min. The entire process was shielded from light, and the cellular morphology was observed and captured using a confocal microscope (CytationM, BioTek, United States).

### 2.10 Quantitative real-time PCR

Total RNA was isolated utilizing TRNzol reagent (Tiangen, China), followed by quantification of concentration and assessment of purity using a Nanodrop nucleic acid and protein analyzer (Thermo Fisher Scientific, United States). After quantifying the RNA concentration, 1,000 ng of the RNA was employed for reverse transcription utilizing the PrimeScript RT Reagent Kit (Takara, China). The RNA expression level was detected using the QuantStudio 3 real-time PCR system (Applied Biosystems, United States) with SYBR Green-based real-time PCR analysis. GAPDH was used as the internal control in this study, and all gene expression levels were calculated using 2^−△△CT^ method. The real-time PCR was conducted according to the following temperature protocol: 95°C for 30 s and then 40 cycles of 95°C for 10 s, 60°C for 20 s, 72°C for 20 s. The RNA primers were procured from Thermo Fisher Scientific (Thermo, China) and the sequences are provided in Supplementary Table S1 in the Supplementary Material.

### 2.11 Western blot analysis

AC16 cardiomyocytes were lysed with 200 μL of RIPA protein lysis buffer per group (Beyotime, China) to extract total protein. Mice myocardial tissue was lysed with 300–400 μL of RIPA buffer to extract total protein. The protein lysates, containing 60 μg of protein, were subjected to SDS-PAGE for separation. Subsequently, they were transferred onto nitrocellulose membranes (Millipore, United States) and subsequently blocked using a solution consisting of 2.5% BSA (Beyotime, China) and 2.5% non-fat milk (BD, United States). The membranes were incubated with the primary antibody overnight at 4°C, followed by a subsequent incubation with the secondary antibody for 1 hour at room temperature on the following day. The immunoblot was tested and quantified by the Image Studio (LI-COR Biosciences, United States). The primary antibodies were used as follows: Atrial natriuretic peptide (ANP, Abcam, ab262703, United Kingdom), Brain natriuretic peptide (BNP, Abcam, ab236101, United Kingdom), NLRP3 (HuaAn Biotechnology, ET1610-93, China), Apoptosis-associated speck-like protein containing a CARD (ASC, Cell Signaling Technology, 67824 and 13833, United States), Caspase-1 (Cell Signaling Technology, 8338, United States), Cas-1 cut (Cell Signaling Technology, 4199, United States), IL-1β (Cell Signaling Technology, 12242, United States), IL-18 (Cell Signaling Technology, 57058 and 54943, United States), and GAPDH (Bioker Biotechnology, BK7021, China).

### 2.12 Statistical analysis

The statistical analyses were conducted using SPSS 22.0, and the results were presented as mean ± SD. Statistical analysis of all data was performed using One-Way ANOVA followed by the Tukey test, with statistical significance defined as *P* < 0.05. The gray value of Western blotting immunobands was analyzed using Odyssey 4.0 software.

## 3 Results

### 3.1 Upregulation of *lncMEG3* in DCM mouse heart and HG-treated AC16 cardiomyocytes

**FIGURE 1:**
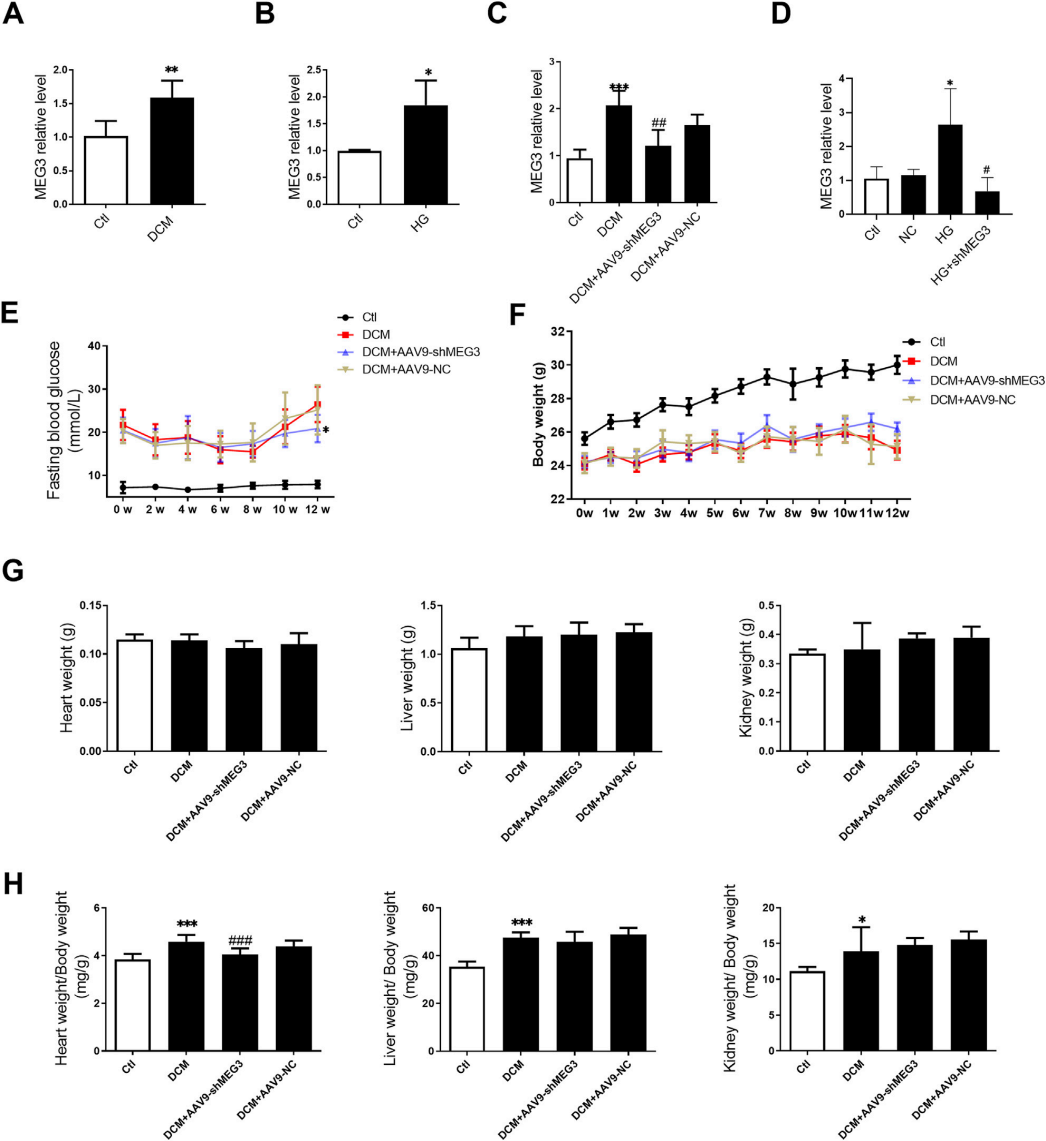
Upregulation of lncMEG3 in DCM mouse heart and HG-treated AC16 cardiomyocytes. **(A)** The lncMEG3 expression was detected by real-time PCR in diabetic mice heart (n = 5, **P < 0.01 vs. Ctl); **(B)** The lncMEG3 expression was detected by real-time PCR in AC16 cardiomyocytes treated with HG (n = 3, *P < 0.05 vs. Ctl); **(C)** The expression of lncMEG3 in DCM injected with AAV9-shMEG3 (n = 4, ***P < 0.001 vs. Ctl, ^##^ *P* < 0.01 vs. DCM); **(D)** The expression of lncMEG3 in AC16 cells treated with shMEG3 transfection (n = 3, ^*^ *P* < 0.05 vs. Ctl, ^#^ *P* < 0.05 vs. HG; **(E)** Temporal changes in fasting blood glucose (FBG) levels over a 12-week period following STZ injection; **(F)** Temporal changes in body weight over a 12-week period following STZ injection; **(G)** The heart weight, liver weight, and kidney weight of mice; **(H)** The heart weight/body weight ratio, liver weight/body weight ratio, and kidney weight/body weight ratio of mice; (n = 10, *P < 0.05, ^***^ *P* < 0.001 vs. Ctl; ^###^ *P* < 0.001 vs. DCM).

To investigate the involvement of *lncMEG3* in DCM, we initially assessed the expression levels of *lncMEG3* in DCM mouse hearts and HG-treated AC16 cardiomyocytes. The expression of *lncMEG3* was significantly upregulated in diabetic mice heart and AC16 cardiomyocytes treated with HG (Figures 1A, B). To further validate the roles of *lncMEG3* in DCM, the interfering RNA molecules targeting *lncMEG3* were synthesized and added into diabetic mice and AC16 cardiomyocytes to assess their inhibitory potential on *lncMEG3* expression. The results demonstrated that shMEG3 exhibited a significant effect (Figures 1C, D).

To elucidate the functional role of *lncMEG3* in DCM, a heart-targeted adeno-associated virus carrying *lncMEG3* interfering RNA (AAV9-shMEG3) was administered via tail-vein injection into diabetic mice. After STZ injection, the mice exhibited an elevated level of fasting blood glucose (FBG) and a reduction in body weight at various time points compared to control mice. The silencing of *lncMEG3* resulted in a significant reduction in fasting blood glucose (FBG) levels at the 12-week time point (Figure 1E). No significant differences were observed in FBG levels at other time points between the diabetic mice and *lncMEG3* knockdown diabetic mice. The knockdown of *lncMEG3* did not result in any statistically significant differences in the body weight of diabetic mice (Figure 1F). No significant differences were observed in heart weight, liver weight, and kidney weight among the mice within each group (Figure 1G). However, compared to the control group, diabetic mice exhibited a significantly increased heart weight/body weight ratio, liver weight/body weight ratio, and kidney weight/body weight ratio. Notably, silencing *lncMEG3* resulted in a significant reduction in the heart weight/body weight of diabetic mice (Figure 1H).

### 3.2 Silencing *lncMEG3* improved cardiac function and ameliorates cardiac remodeling of DCM

**FIGURE 2:**
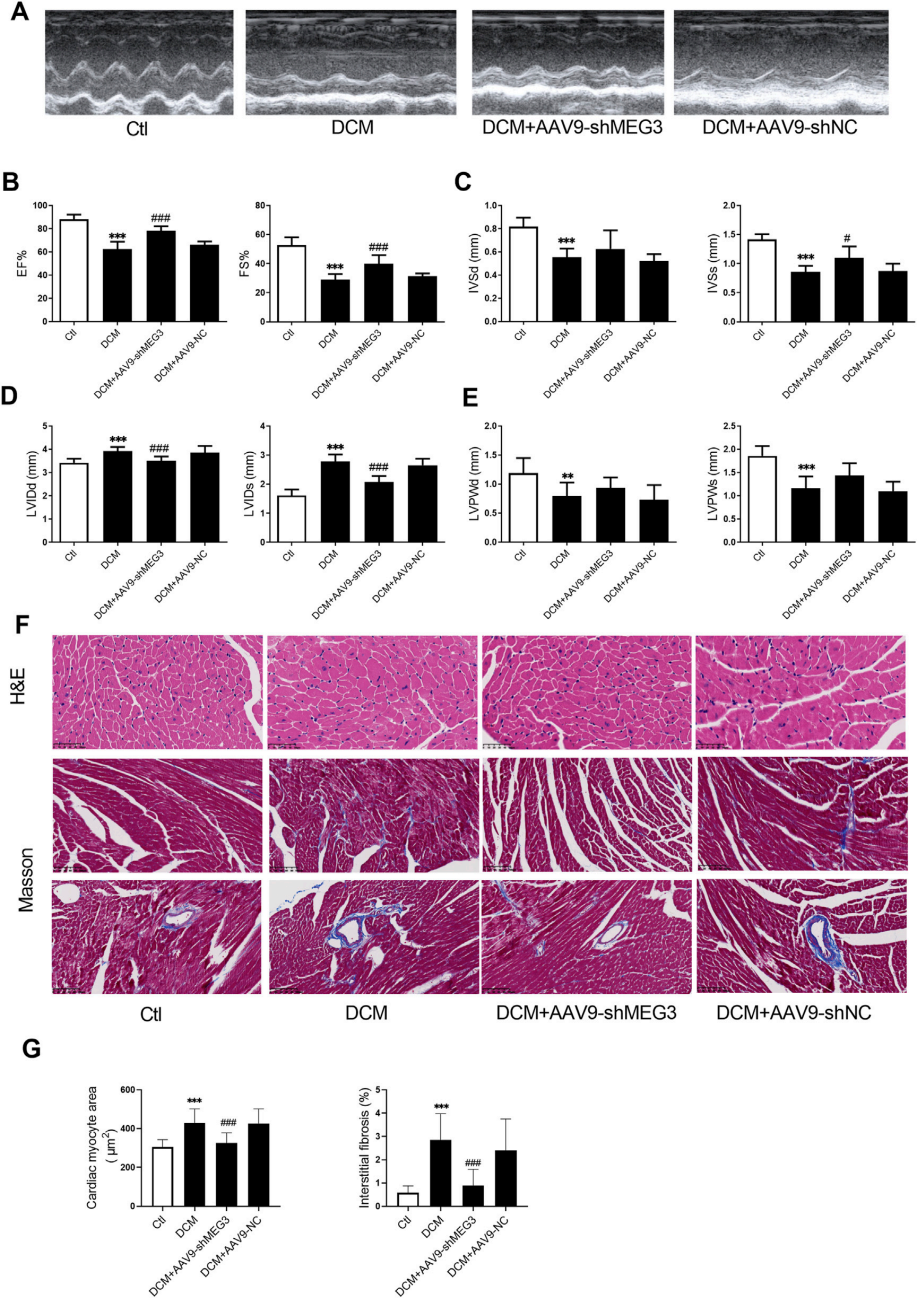
Silencing lncMEG3 improved cardiac function and ameliorates cardiac remodeling of DCM. **(A)** Representative images of mice cardiac echocardiography. **(B)** Ejection fraction (EF) and Fractional shortening (FS), **(C)** left ventricular end-diastolic septal thickness (IVSd) and left ventricular end-systolic septal thickness (IVSs), **(D)** left ventricular end-diastole internal dimension (LVIDd) and left ventricular end-systolic internal dimension (LVIDs), **(E)** left ventricular end-diastole posterior wall thickness (LVPWd) and left ventricular end-systolic posterior wall thickness (LVPWs) were evaluated by echocardiography in different treatment groups. Date were expressed as means ± SD, n = 10 mice per group. **P < 0.01, ***P < 0.001 vs. Ctl, ^#^ *P* < 0.05, ^###^ *P* < 0.001 vs. DCM. **(F)** Representative images depicting histological sections stained with hematoxylin and eosin (H&E) for myocardial hypertrophy (Magnification, X400), as well as Masson’s trichrome staining for interstitial fibrosis and perivascular fibrosis (Magnification, X200). **(G)** Quantitative analysis of cardiomyocyte area and extent of interstitial fibrosis, n = 3, ***P < 0.001 vs. Ctl; ^###^ *P* < 0.001 vs. DCM.

Echocardiography was conducted on mice to investigate the impact of *lncMEG3* silencing on cardiac function in diabetic murine models. The results showed that ejection fraction (EF), fractional shortening (FS), left ventricular end-diastolic septal thickness (IVSd), left ventricular end-systolic septal thickness (IVSs), left ventricular end-diastole internal dimension (LVIDd), left ventricular end-systolic internal dimension (LVIDs), left ventricular end-diastole posterior wall thickness (LVPWd), and left ventricular end-systolic posterior wall thickness (LVPWs) were significantly deteriorated in diabetic mice compared with the control group (Figures 2A–E). However, silencing of *lncMEG3* mitigated the decline in EF, FS, and IVSs, while preventing the increase in LVIDs and LVIDd in diabetic mice (Figures 2A–D). To investigate the impact of *lncMEG3* silencing on cardiomyocyte hypertrophy and cardiac fibrosis in diabetic mice, we employed H&E staining and Masson staining to examine pathological alterations in murine hearts. The diabetic group exhibited a significant increase in the area of cardiomyocytes, myocardial interstitial fibrosis, and perivascular fibrosis, as depicted in Figures 2F,G. Conversely, silencing *lncMEG3* expression in cardiomyocytes of mice remarkably reduced the aforementioned areas in diabetic mice. Notably, the negative control group did not exert a significant effect on cardiac remodeling.

### 3.3 Silencing *lncMEG3* improved myocardial hypertrophy in DCM

**FIGURE 3:**
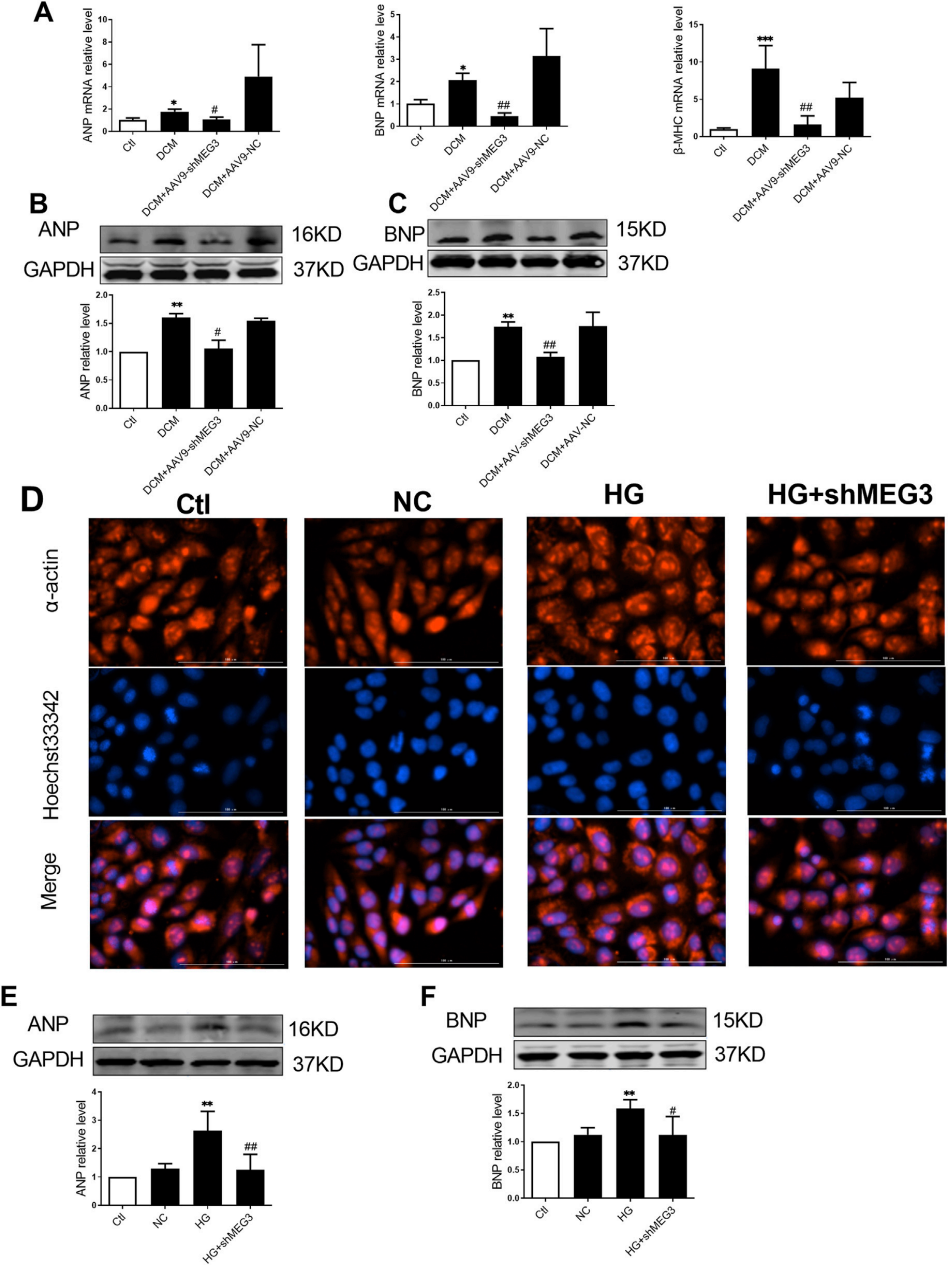
Silencing lncMEG3 improved myocardial hypertrophy in DCM. The relative mRNA levels of **(A)** Atrial natriuretic peptide (ANP), brain natriuretic peptide (BNP) and β-myosin heavy chain (β-MHC) were measured by real-time PCR. Protein expression level of **(B)** Atrial natriuretic peptide (ANP) and **(C)** brain natriuretic peptide (BNP) of mouse hearts were detected by Western blotting in different groups, n = 4 per group. Data were expressed as means ± SD, *P < 0.05, **P < 0.01, ***P < 0.001 vs. Ctl, ^#^ *P* < 0.05, ^##^ *P* < 0.01 vs. DCM. **(D)** AC16 cardiomyocytes representative images of immunofluorescence staining on hypertrophy level. Magnification, X400, n = 3 per group. The protein expression level of **(E)** Atrial natriuretic peptide (ANP) and **(F)** brain natriuretic peptide (BNP) of AC16 cardiomyocytes were detected by Western blotting in different groups, n = 4 per group. Data were expressed as means ± SD, **P < 0.01 vs. Ctl, ^#^ *P* < 0.05, ^##^ *P* < 0.01 vs. HG.

To investigate the effect of *lncMEG3* on DCM, we assessed the level of myocardial hypertrophy-associated proteins in DCM mouse heart. ANP, BNP and β-MHC were utilized as markers of cardiac hypertrophy to evaluate the occurrence of myocardial hypertrophy in DCM mice. Our findings revealed a significant upregulation of ANP, BNP, and β-MHC expression in DCM mice. Moreover, *lncMEG3* knockdown reduced the levels of myocardial hypertrophy-related genes (Figures 3A–C). Then, we explored the regulation of *lncMEG3* on cardiac hypertrophy induced by HG *in vitro*. The results demonstrated that myocardial cell area was increased in HG-treated AC16 cardiomyocytes, with elevated protein levels of ANP and BNP. However, knockdown of *lncMEG3* ameliorated these phenomena (Figures 3D–F), indicating that *lncMEG3* deficiency significantly attenuated the deterioration of myocardial hypertrophy in DCM.

### 3.4 Silencing *lncMEG3* inhibits pyroptosis signaling pathway in DCM mouse heart

**FIGURE 4:**
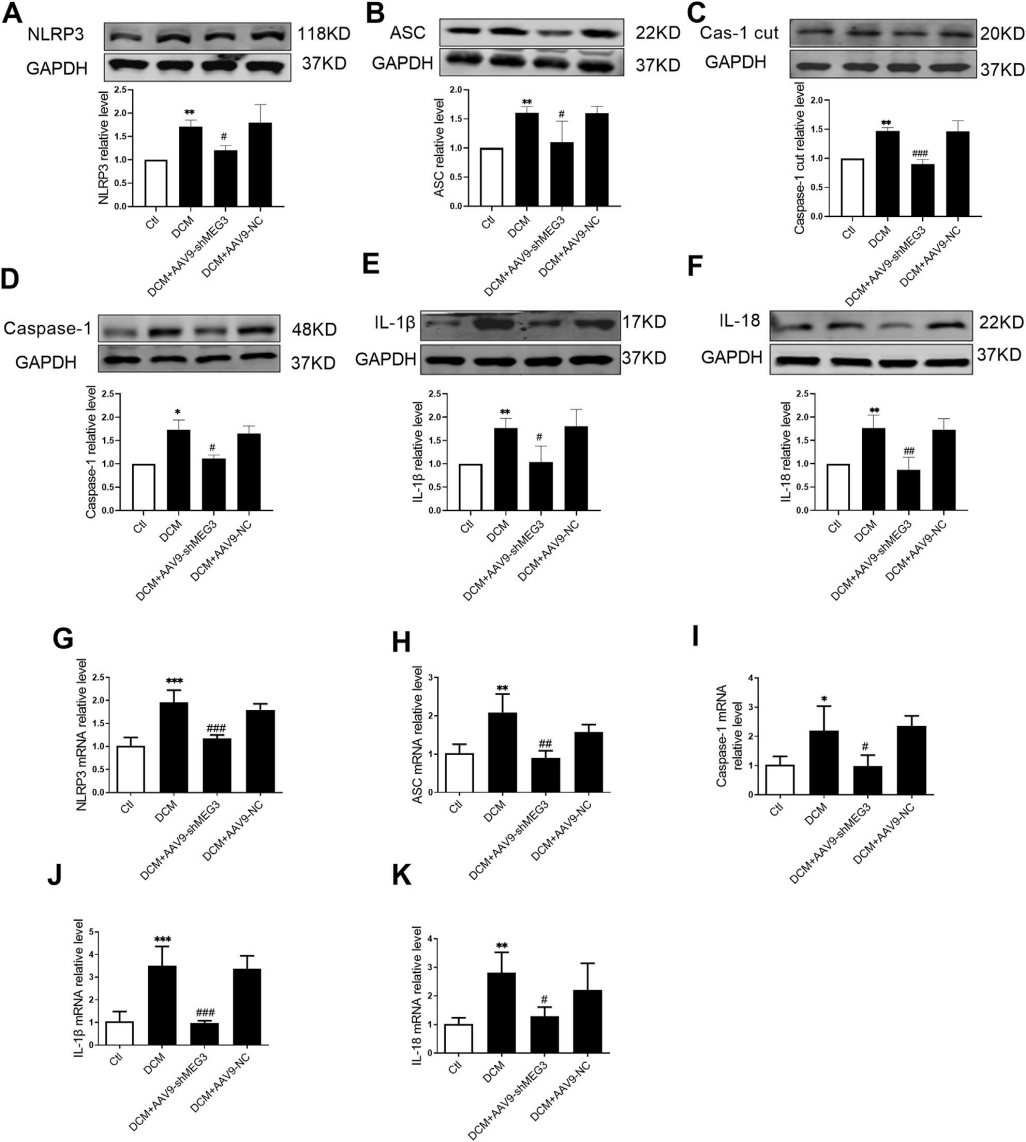
Silencing lncMEG3 inhibits pyroptosis signaling pathway in DCM mouse heart. The protein expression level of **(A)** NLRP3, **(B)** ASC, **(C)** Cleaverd caspase-1, **(D)** Caspase-1, **(E)** IL-1β, and **(F)** IL-18 were detected by Western blotting analysis, n = 4 per group. The relative mRNA levels of **(G)** NLRP3, **(H)** ASC, **(I)** Caspase-1, **(J)** IL-1β, and **(K)** IL-18 were detected by real-time PCR. n = 4 per group, data were expressed as means ± SD, *P < 0.05, **P < 0.01, ***P < 0.001 vs. Ctl, ^#^ *P* < 0.05, ^##^ *P* < 0.01, ^###^ *P* < 0.001 vs. DCM.

The impact of *lcnMEG3* deficiency on myocardial pyroptosis was investigated by assessing the expression levels of key factors associated with the NLRP3/Caspase-1/IL-1β classical pyroptosis signaling pathway in murine cardiac tissue. As shown in Figures 4A–F, the protein expressions of NLRP3, ASC, Caspase-1 cut, Caspase-1, IL-1β, and IL-18 were found to be elevated in the hearts of diabetic mice compared to the control group. While, the silencing *lncMEG3* resulted in a significant reduction in expression levels of pyroptosis signaling factors. The mRNA levels of NLRP3, ASC, Caspase-1, IL-1β, and IL-18 in the heart tissues of different mouse groups were quantified through real-time PCR. It was found that compared with the DCM group, silencing *lncMEG3* significantly attenuated the mRNA levels of NLRP3, ASC, Caspase-1, IL-1β, and IL-18 in the hearts of diabetic mice (Figures 4G–K). These findings suggest that silencing *lncMEG3* can effectively inhibit the pyroptosis signaling pathway in diabetic mouse hearts.

### 3.5 Silencing *lncMEG3* attenuates pyroptosis signaling pathway in high glucose-treated AC16 cardiomyocytes

**FIGURE 5:**
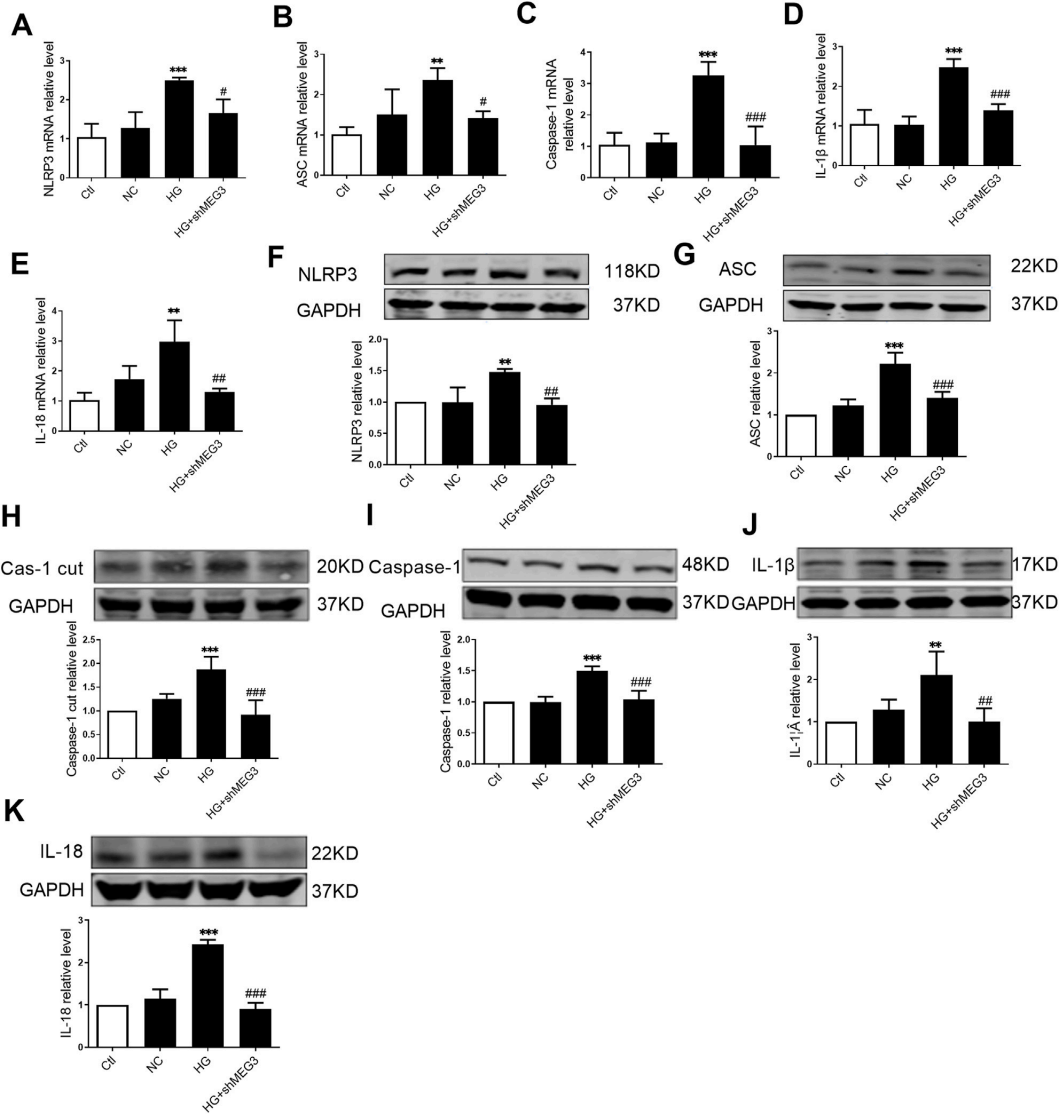
Silencing lncMEG3 attenuates pyroptosis signaling pathway in high glucose-treated AC16 cardiomyocytes. The relative mRNA levels of **(A)** NLRP3, **(B)** ASC, **(C)** Caspase-1, **(D)** IL-1β, and **(E)** IL-18 were measured by real-time PCR in AC16 cardiomyocytes treated with HG and shMEG3, n = 3 to 4 per group. The protein levels **(F)** NLRP3, **(G)** ASC, **(H)** Cleaved caspase-1, **(I)** Caspase-1, **(J)** IL-1β, and **(K)** IL-18 were detected by Western blotting in AC16 cardiomyocytes treated with HG and shMEG3, n = 4 per group. Data were expressed as means ± SD, **P < 0.01, ***P < 0.001 vs. Ctl, ^#^ *P* < 0.05, ^##^ *P* < 0.01, ^###^ *P* < 0.001 vs. HG.

We subsequently investigated the effect of *lncMEG3* on pyroptosis signaling pathway treated by HG (35 mmol/L) *in vitro*. The exposure to HG significantly facilitated activation of pyroptosis signaling pathway. The level of *lncMEG3* was reduced in cultured AC16 cardiomyocytes through transfection with *lncMEG3* shRNA. *LncMEG3* knockdown resulted in a decrease in the mRNA and protein levels of NLRP3, ASC, Caspase-1, IL-1β, and IL-18 (Figure 5), indicating that silencing *lncMEG3* effectively inhibit the activation of pyroptosis signaling pathway in AC16 cardiomyocytes.

### 3.6 *LncMEG3* is as a ceRNA for *miR-223* to regulate the myocardial hypertrophy in DCM

**FIGURE 6:**
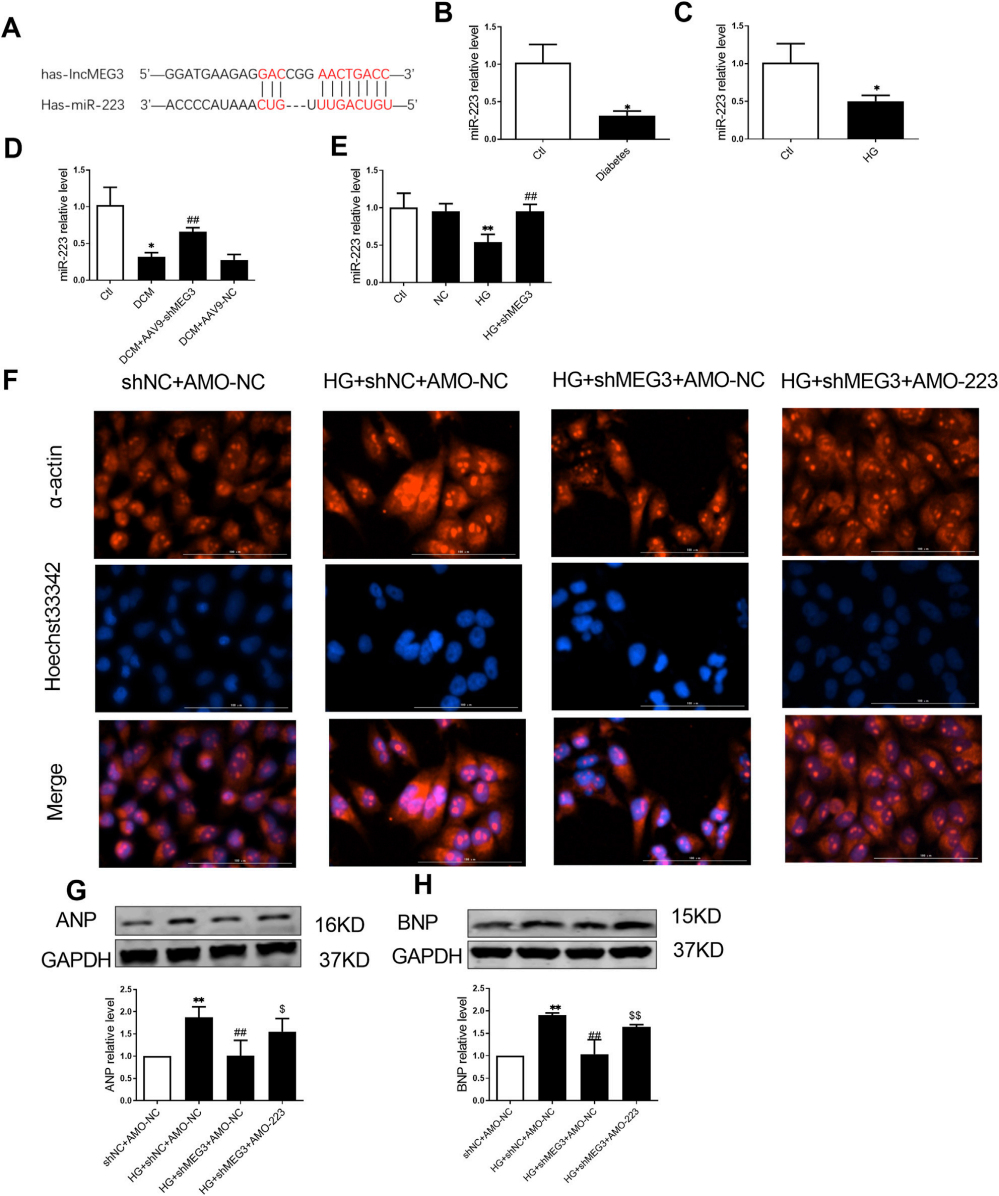
LncMEG3 is as a ceRNA for miR-223 to regulate the myocardial hypertrophy in DCM. **(A)** Bioinformatics prediction and analysis of the binding site between LncMEG3 and miR-223. The relative level of miR-223 of **(B)** diabetic mice heart and **(C)** AC16 cardiomyocytes treated with HG were measured by real-time PCR, n = 3 to 4 per group. Data were expressed as means ± SD, *P < 0.05 vs. Ctl. **(D)** Level of miR-223 in the cardiac tissue of mice were measured by real-time PCR in different groups, n = 4 per group. Data were expressed as means ± SD, ^*^ *P* < 0.05 vs. Ctl, ^##^ *P* < 0.01 vs. DCM. **(E)** Level of miR-223 in different groups of AC16 cardiomyocytes were measured by real-time PCR, n = 4 per group. Data were expressed as means ± SD, **P < 0.01 vs. Ctl; ^##^ *P* < 0.01 vs. HG. **(F)** Representative images of immunofluorescence staining for the detection of hypertrophy level in AC16 cardiomyocytes after co-transfection with lncMEG3 interfering RNA and miR-223 inhibitor (AMO-223). Magnification, ×400, n = 3. The protein expression level of **(G)** Atrial natriuretic peptide (ANP) and **(H)** brain natriuretic peptide (BNP) of AC16 cardiomyocytes after co-transfection with lncMEG3 interfering RNA and miR-223 inhibitor (AMO-223) were detected by Western blotting, n = 3 to 4 per group. Data were expressed as means ± SD, **P < 0.01 vs. shNC + AMO-NC, ^##^ *P* < 0.01 vs. HG + shNC + AMO-NC, ^$^ *P* < 0.05, ^$$^ *P* < 0.01 vs. HG + shMEG3 +AMO-NC.

We next continued to explore how *lncMEG3* regulate pyroptosis signaling pathway in HG-treated AC16 cardiomyocytes. Through RNAhybrid web analysis, we predicted potential microRNA targets of *lncMEG3* and identified a binding site for *miR-223* within lncMEG3 (Figure 6A). We quantified the expression levels of *miR-223* through real-time PCR and observed a significant decrease in *miR-223* RNA levels in DCM mouse and AC16 cardiomyocytes treated with HG (Figures 6B, C). *LncMEG3* shRNA dramatically increased the expression of *miR-223* (Figures 6D, E). Several studies have reported that NLRP3 expression is regulated by *miR-223* (Zhang et al., 2018).

*LncMEG3* shRNA and AMO-223 (*miR-223* inhibitor) were co-transfected into AC16 cardiomyocytes to further elucidate the role of *lncMEG3* in promoting cardiomyocyte hypertrophy by regulating *miR-223*. Therefore, α-acting staining and assessment of hypertrothy-related gene expression were used to evaluate myocardial hypertrophy. As shown in Figures 6F–H, the area of cardiomyocytes and the expressions of ANP and BNP were significantly upregulated by AMO-223. These findings suggest that *lncMEG3* may promote myocardial hypertrophy by inhibiting the level of *miR-223*.

### 3.7 *LncMEG3* promotes pyroptosis signaling pathway in in high glucose-treated AC16 cardiomyocytes through *miR-223*/NLRP3 axis

**FIGURE 7:**
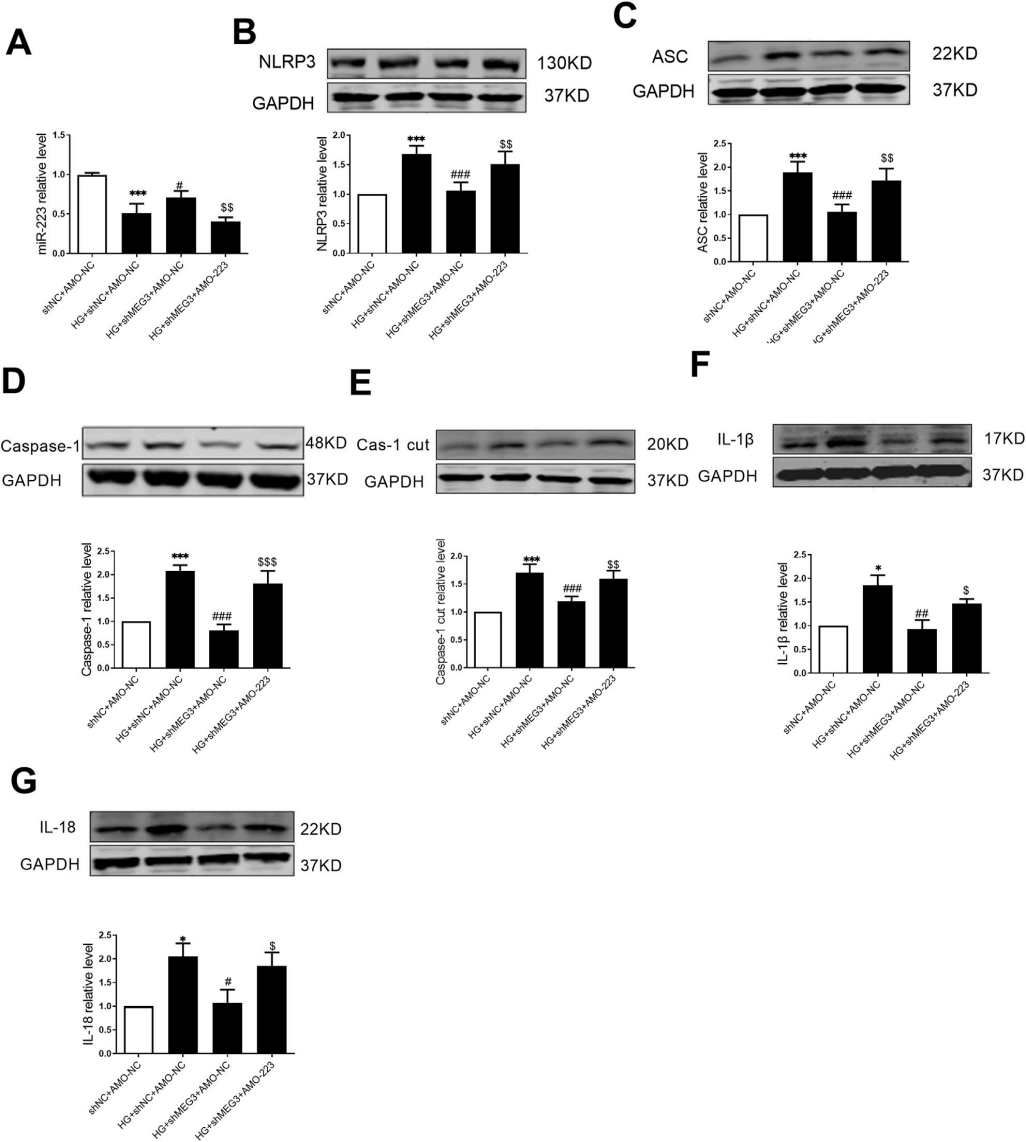
LncMEG3 promotes pyroptosis signaling pathway in high glucose-treated AC16 cardiomyocytes through miR-223/NLRP3 axis. **(A)** The relative level of miR-223 of AC16 cardiomyocytes after co-transfection with lncMEG3 interfering RNA and miR-223 inhibitor (AMO-223) was measured by real-time PCR. The protein expression level of **(B)** NLRP3, **(C)** ASC, **(D)** Caspase-1, **(E)** Cleaved caspase-1, **(F)** IL-1β and **(G)** IL-18 of AC16 cardiomyocytes after co-transfection with lncMEG3 interfering RNA and miR-223 inhibitor (AMO-223) were detected by Western blotting. n = 4 per group, data were expressed as means ± SD, *P < 0.05, ***P < 0.001 vs. shNC + AMO-NC, ^#^ *P* < 0.05, ^##^ *P* < 0.01, ^###^ *P* < 0.001 vs. HG + shNC + AMO-NC, ^$^ *P* < 0.05, ^$$^ *P* < 0.01, ^$$$^ *P* < 0.001 vs. HG + shMEG3 +AMO-NC.

To elucidate the involvement of *lncMEG3*/*miR-223* in the regulation of the NLRP3 inflammasome-mediated pyroptosis signaling pathway, we employed a co-transfection approach to inhibit the *lncMEG3*/*miR-223* axis by transfecting *lncMEG3* shRNA and AMO-223 into AC16 cardiomyocytes under high glucose conditions. The real-time PCR analysis revealed that the expression of *miR-223* in AC16 cardiomyocytes stimulated by HG was significantly downregulated compared to the negative control group. However, knockdown of *lncMEG3* in HG-treated AC16 cardiomyocytes resulted in a significant upregulation of *miR-223* expression. Conversely, co-transfection of *lncMEG3* shRNA and AMO-223 led to a reduction in *miR-223* expression. The protein expression levels of factors associated with the NLRP3 inflammasome-mediated pyroptosis signaling pathway in AC16 cells were assessed by Western blotting. As depicted in Figure 7, compared to the HG group, knockdown of *lncMEG3* shRNA and stimulation with HG significantly decreased the expressions of pyroptosis-related proteins including NLRP3, ASC, Caspase-1, Caspase-1 cut, IL-1β, and IL-18. Conversely, co-transfection of *lncMEG3* shRNA and AMO-223 upregulated the expression of these pyroptosis-related proteins. These findings suggest that silencing *lncMEG3* inhibits the cell pyroptosis pathway through *miR-223* upregulation, thereby ameliorating diabetic cardiomyopathy.

## 4 Discussion

The pathophysiological changes associated with DCM encompass myocardial hypertrophy, collagen deposition, cardiac dysfunction, inflammation, increased ROS levels, and cellular apoptosis. However, the intricate and unexplained impact of lncRNAs on these pathophysiological changes in DCM remains to be elucidated. In this study, we established a type 1 diabetic model to investigate the involvement of *lncMEG3* and NLRP3 inflammasome-mediated pyroptosis mechanisms in DCM.

Recent studies have substantiated the pivotal role of lncRNAs as crucial regulatory molecules in the pathological process of cardiovascular disease. Ablation of lncRNA *MIAT* confers cardioprotection against angiotensin Ⅱ-induced cardiac hypertrophy and transverse aortic constriction-induced heart failure through augmenting calcium handling and contractility in cardiomyocytes (Yang et al., 2021). LncRNA *LIPTER* overexpression attenuates cardiac lipotoxicity, enhances cardiac function, and ameliorates cardiac remodeling in high-fat-diat-fed mice (Han et al., 2023). Piccoli et al. (2017) demonstrated a crucial role of in promoting cardiac fibrosis, and the knockdown of *lncMEG3* prevents both cardiac fibrosis and diastolic dysfunction.

The expression of *LncMEG3* is highly abundant in cardiac tissues and it plays a crucial role in the regulation of various cardiac diseases, including viral myocarditis (Xue et al., 2020) and heart failure (Mi et al., 2023). In mouse myocardial infarction injury, *lncMEG3* was directly upregulated by *P53* under hypoxic conditions, thereby facilitating myocardial cell apoptosis (Wu et al., 2018). In our study, we observed an upregulation of *lncMEG3* expression in the hearts of diabetic mice and AC16 cardiomyocytes treated with HG. Suppression of *lncMEG3* level exhibited ameliorative effects on myocardial hypertrophy, cardiac fibrosis, cardiac dysfunction, and heart weight/body weight ratio in diabetic mice.

Silencing of *lncMEG3* ameliorated cardiac dysfunction in diabetic cardiomyopathy, which was associated with a concomitant reduction in FBG levels. The improved cardiac function could improve blood perfusion to peripheral tissues such as skeletal muscle, adipose tissue, and the liver. This enhanced perfusion facilitates systemic metabolic processes, including glucose delivery and uptake (Cugusi et al., 2015). Several studies have demonstrated that the plasma levels of *lncMEG3* were significantly upregulated in patients with diabetes mellitus compared to healthy controls (Alrefai et al., 2023; Heydari et al., 2023). The upregulation of *lncMEG3* exacerbates hepatic insulin resistance by increasing FOXO1 expression (Zhu et al., 2019). Conversely, downregulation of *lncMEG3* could lower fasting blood glucose (FBG) levels and mitigate insulin resistance (Zhu et al., 2019). In our study, we also found that *lncMEG3* knockdown by AAV9-shMEG3 could reducing FBG levels at the 12-week time point. It is possible that a small amount of AAV9-shMEG3 decreases *lncMEG3* expression in tissues such as the liver, thereby producing hypoglycemic effects.

Increasing evidence has revealed the presence of pyroptosis in various cardiovascular diseases, including diabetic cardiomyopathy (Meng et al., 2022), myocardial infarction (Wang et al., 2022), myocardial ischemia-reperfusion (Zheng et al., 2022), endothelial cell dysfunction (Zheng et al., 2022), and heart failure (Chai et al., 2022). Pyroptosis is an inflammatory programmed cell death characterized by the activation of the NLRP3 inflammasome, which triggers a cascade of cellular events such as cell swelling, pore formation in the plasma membrane, membrane rupture, increased permeability, DNA fragmentation, release of intracellular contents and inflammatory mediators, culminating in a robust inflammatory response (Lu et al., 2020). It is well established that pyroptosis plays a crucial role in the pathogenesis of DCM (Zeng et al., 2019). Studies have demonstrated that silencing NLRP3 can inhibit pyroptosis in H9c2 cardiomyocytes exposed to high-glucose conditions and ameliorate cardiac pyroptosis, inflammation, and fibrosis in Type 2 diabetes mellitus (T2DM) rats (Luo et al., 2014; Yang et al., 2018). By downregulating the expression level of miR-30d, high glucose-induced myocardial pyroptosis can be significantly inhibited, thereby mitigating the inflammatory response and enhancing cardiac function (Li et al., 2014). However, the precise mechanisms underlying the initiation and regulation of pyroptosis mediated by the NLRP3 inflammasome complex, which triggers caspase-1 activation in DCM, remain poorly elucidated. Furthermore, the effect of *lncMEG3* in activation or regulation of NLRP3 inflammasome-mediated pyroptosis is being acknowledged in other physiological and pathological process. The expression of *lncMEG3* was upregulated in lipopolysaccharide (LPS)-induced microglial inflammation, leading to the activation of NLRP3/caspase-1 signaling pathway. Conversely, downregulation of *lncMEG3* significantly attenuated NLRP3 inflammasome activation (Meng et al., 2021). However, the cellular mechanisms of *lncMEG3* in DCM remain, which perhaps as a crucial therapeutic target.

Here, we observed an upregulation of NLRP3 inflammasome-associated proteins including NLRP3, ASC, caspase-1 cut, IL-1β, and IL-18 in DCM. Importantly, inhibition of *lncMEG3* expression significantly attenuated the activation of the NLRP3 inflammasome-mediated pyroptosis pathway.

LncRNAs have been discovered to regulate biological functions and pathological processes through different mechanisms. They modulate gene expression by functioning as microRNA sponges. He et al. (2017) demonstrated that lncMEG3 acts as a competing endogenous RNA (ceRNA) by binding *miR-9*, thereby modulating the phenotype of *lncMEG3*-mediated vascular endothelial cells. The downregulation of *miR-223* in the endothelium of HFD mice has been demonstrated, while *lncMEG3* acts as a sponge for *miR-223* to enhance the expression of NLRP3-inflammasome in the endothelium of HFD mice (Zhang et al., 2018). In our study, bioinformatics predictions revealed that *miR-223* exhibits binding sites for both *lncMEG3* and NLRP3. In this study, the hypertrophy level of HG-stimulated AC16 cells co-transfected with shMEG3 and AMO-223 to silence *lncMEG3* and inhibit *miR-223* was significantly increased compared to cells transfected with shMEG3 alone, accompanied by upregulated expression of NLRP3 and its downstream inflammatory factors. The regulatory role of *lncMEG3* in NLRP3 inflammasome-mediated pyroptosis pathway through *miR-223* targeting has been demonstrated, suggesting the potential of *lncMEG3* possibly as a novel therapeutic target for managing DCM by modulating *miR-223*.

Our data validate *lncMEG3* as a therapeutically actionable target; however, the specificity of targeting *lncMEG3* in the heart requires further improvement. In our study, we utilized cardiac-targeted AAV9-delivered shRNA to suppress *lncMEG3* expression, but this approach lacks sufficient specificity for cardiac tissues. The application of CRISPR/Cas9 gene knockout technology could potentially enhance cardiac specificity more effectively.

In conclusion, our findings demonstrate the upregulation of *lncMEG3* in the hearts of DCM mouse models and HG-induced AC16 cardiomyocytes. Silencing *lncMEG3* exhibits potential for ameliorating myocardial hypertrophy and pyroptosis through modulation of the *miR-223*/NLRP3/caspase-1 pathway. This study provides novel insights into the therapeutic effect and underlying mechanism of *lncMEG3* in improving DCM myocardial function, offering a promising avenue for preventing and treating DCM.

## Data Availability

The original contributions presented in the study are included in the article/Supplementary Material, further inquiries can be directed to the corresponding authors.

## References

[B1] Alrefai A. A. Khader H. F. Elbasuony H. A. Elzorkany K. M. Saleh A. A. (2023). Evaluation of the expression levels of lncRNAs H19 and MEG3 in patients with type 2 diabetes mellitus. Mol. Biol. Rep. 50, 6075–6085. 10.1007/s11033-023-08569-0 37294471

[B2] Bridges M. C. Daulagala A. C. Kourtidis A. (2021). LNCcation: lncRNA localization and function. J. Cell Biol. 220, e202009045. 10.1083/jcb.202009045 33464299 PMC7816648

[B3] Chai R. Xue W. Shi S. Zhou Y. Du Y. Li Y. (2022). Cardiac remodeling in heart failure: role of pyroptosis and its therapeutic implications. Front. Cardiovasc Med. 9, 870924. 10.3389/fcvm.2022.870924 35509275 PMC9058112

[B4] Chen R. Zhang G. Sun K. Chen A. F. (2024). Aging-associated ALKBH5-m6A modification exacerbates doxorubicin-induced cardiomyocyte apoptosis via AT-rich interaction domain 2. J. Am. Heart Assoc. 13, e031353. 10.1161/JAHA.123.031353 38156523 PMC10863816

[B5] Cugusi L. Cadeddu C. Nocco S. Orrù F. Bandino S. Deidda M. (2015). Effects of an aquatic-based exercise program to improve cardiometabolic profile, quality of life, and physical activity levels in men with type 2 diabetes mellitus. PM R. 7, 141–148. 10.1016/j.pmrj.2014.09.004 25217820

[B6] Denes A. Lopez-Castejon G. Brough D. (2012). Caspase-1: is IL-1 just the tip of the ICEberg? Cell Death Dis. 3, e338. 10.1038/cddis.2012.86 22764097 PMC3406585

[B7] Deng B. Hu Y. Sheng X. Zeng H. Huo Y. (2020). miR-223-3p reduces high glucose and high fat-induced endothelial cell injury in diabetic mice by regulating NLRP3 expression. Exp. Ther. Med. 20, 1514–1520. 10.3892/etm.2020.8864 32765674 PMC7388564

[B8] Dillmann W. H. (2019). Diabetic cardiomyopathy. Circ. Res. 124, 1160–1162. 10.1161/circresaha.118.314665 30973809 PMC6578576

[B9] Han L. Huang D. Wu S. Liu S. Wang C. Sheng Y. (2023). Lipid droplet-associated lncRNA LIPTER preserves cardiac lipid metabolism. Nat. Cell Biol. 25, 1033–1046. 10.1038/s41556-023-01162-4 37264180 PMC10344779

[B10] He C. Yang W. Yang J. Ding J. Li S. Wu H. (2017). Long noncoding RNA*MEG3*Negatively regulates proliferation and angiogenesis in vascular endothelial cells. DNA Cell Biol. 36, 475–481. 10.1089/dna.2017.3682 28418724

[B11] Heydari N. Sharifi R. Nourbakhsh M. Golpour P. Nourbakhsh M. (2023). Long non-coding RNAs TUG1 and MEG3 in patients with type 2 diabetes and their association with endoplasmic reticulum stress markers. J. Endocrinol. Invest. 46, 1441–1448. 10.1007/s40618-023-02007-5 36662419

[B12] Holdt L. M. Teupser D. (2012). Recent studies of the human chromosome 9p21 locus, which is associated with atherosclerosis in human populations. Arterioscler. Thromb. Vasc. Biol. 32, 196–206. 10.1161/atvbaha.111.232678 22258902

[B13] Li X. Du N. Zhang Q. Li J. Chen X. Liu X. (2014). MicroRNA-30d regulates cardiomyocyte pyroptosis by directly targeting foxo3a in diabetic cardiomyopathy. Cell Death Dis. 5, e1479. 10.1038/cddis.2014.430 25341033 PMC4237254

[B14] Li X. Zhao J. Geng J. Chen F. Wei Z. Liu C. (2019). Long non‐coding RNA MEG3 knockdown attenuates endoplasmic reticulum stress‐mediated apoptosis by targeting p53 following myocardial infarction. J. Cell Mol. Med. 23, 8369–8380. 10.1111/jcmm.14714 31631486 PMC6850962

[B15] Liu C. Yao Q. Hu T. Cai Z. Xie Q. Zhao J. (2022). Cathepsin B deteriorates diabetic cardiomyopathy induced by streptozotocin via promoting NLRP3-mediated pyroptosis. Mol. Ther. Nucleic Acids 30, 198–207. 10.1016/j.omtn.2022.09.019 36250207 PMC9554743

[B16] Lu F. Lan Z. Xin Z. He C. Guo Z. Xia X. (2020). Emerging insights into molecular mechanisms underlying pyroptosis and functions of inflammasomes in diseases. J. Cell Physiol. 235, 3207–3221. 10.1002/jcp.29268 31621910 PMC13482838

[B17] Luo B. Li B. Wang W. Liu X. Xia Y. Zhang C. (2014). NLRP3 gene silencing ameliorates diabetic cardiomyopathy in a type 2 diabetes rat model. PLOS ONE 9, e104771. 10.1371/journal.pone.0104771 25136835 PMC4138036

[B18] Meng J. Ding T. Chen Y. Long T. Xu Q. Lian W.-Q. (2021). LncRNA-Meg3 promotes Nlrp3-mediated microglial inflammation by targeting miR-7a-5p. Int. Immunopharmacol. 90, 107141. 10.1016/j.intimp.2020.107141 33189612

[B19] Meng L. Lin H. Huang X. Weng J. Peng F. Wu S. (2022). METTL14 suppresses pyroptosis and diabetic cardiomyopathy by downregulating TINCR lncRNA. Cell Death Dis. 13, 38. 10.1038/s41419-021-04484-z 35013106 PMC8748685

[B20] Mi S. Huang F. Jiao M. Qian Z. Han M. Miao Z. (2023). Inhibition of MEG3 ameliorates cardiomyocyte apoptosis and autophagy by regulating the expression of miRNA-129-5p in a mouse model of heart failure. Redox Rep. 28, 2224607. 10.1080/13510002.2023.2224607 37338021 PMC10286679

[B21] Nakamura K. Miyoshi T. Yoshida M. Akagi S. Saito Y. Ejiri K. (2022). Pathophysiology and treatment of diabetic cardiomyopathy and heart failure in patients with diabetes mellitus. Int. J. Mol. Sci. 23, 3587. 10.3390/ijms23073587 35408946 PMC8999085

[B22] Piccoli M. T. Gupta S. K. Viereck J. Foinquinos A. Samolovac S. Kramer F. L. (2017). Inhibition of the cardiac fibroblast-enriched lncRNA Meg3 prevents cardiac fibrosis and diastolic dysfunction. Circ. Res. 121, 575–583. 10.1161/CIRCRESAHA.117.310624 28630135

[B23] Qu X. Du Y. Shu Y. Gao M. Sun F. Luo S. (2017). MIAT is a pro-fibrotic long non-coding RNA governing cardiac fibrosis in post-infarct myocardium. Sci. Rep. 7, 42657. 10.1038/srep42657 28198439 PMC5309829

[B24] Saeedi P. Salpea P. Karuranga S. Petersohn I. Malanda B. Gregg E. W. (2020). Mortality attributable to diabetes in 20-79 years old adults, 2019 estimates: results from the International Diabetes Federation Diabetes Atlas, 9th edition. Diabetes Res. Clin. Pract. 162, 108086. 10.1016/j.diabres.2020.108086 32068099

[B25] Vande Walle L. Lamkanfi M. (2020). Snapshot of a deadly embrace: the caspase-1-GSDMD interface. Immunity 53, 6–8. 10.1016/j.immuni.2020.06.019 32668229

[B26] Viereck J. Kumarswamy R. Foinquinos A. Xiao K. Avramopoulos P. Kunz M. (2016). Long noncoding RNA *Chast* promotes cardiac remodeling. Sci. Transl. Med. 8, 326ra22. 10.1126/scitranslmed.aaf1475 26888430

[B27] Wang Y.-W. Dong H.-Z. Tan Y.-X. Bao X. Su Y.-M. Li X. (2022). HIF-1α-regulated lncRNA-TUG1 promotes mitochondrial dysfunction and pyroptosis by directly binding to FUS in myocardial infarction. Cell Death Discov. 8, 178. 10.1038/s41420-022-00969-8 35396503 PMC8993815

[B28] Wu H. Zhao Z.-A. Liu J. Hao K. Yu Y. Han X. (2018). Long noncoding RNA Meg3 regulates cardiomyocyte apoptosis in myocardial infarction. Gene Ther. 25, 511–523. 10.1038/s41434-018-0045-4 30287867

[B29] Xue Y.-L. Zhang S.-X. Zheng C.-F. Li Y.-F. Zhang L.-H. Su Q.-Y. (2020). Long non-coding RNA MEG3 inhibits M2 macrophage polarization by activating TRAF6 via microRNA-223 down-regulation in viral myocarditis. J. Cell Mol. Med. 24, 12341–12354. 10.1111/jcmm.15720 33047847 PMC7686963

[B30] Yan M. Li Y. Luo Q. Zeng W. Shao X. Li L. (2022). Mitochondrial damage and activation of the cytosolic DNA sensor cGAS–STING pathway lead to cardiac pyroptosis and hypertrophy in diabetic cardiomyopathy mice. Cell Death Discov. 8, 258. 10.1038/s41420-022-01046-w 35538059 PMC9091247

[B31] Yang F. Qin Y. Lv J. Wang Y. Che H. Chen X. (2018). Silencing long non-coding RNA Kcnq1ot1 alleviates pyroptosis and fibrosis in diabetic cardiomyopathy. Athy. Cell Death Dis. 9, 1000. 10.1038/s41419-018-1029-4 PMC6155223 30250027

[B32] Yang L. Deng J. Ma W. Qiao A. Xu S. Yu Y. (2021). Ablation of lncRNA *Miat* attenuates pathological hypertrophy and heart failure. Theranostics 11, 7995–8007. 10.7150/thno.50990 34335976 PMC8315059

[B33] Zeng C. Wang R. Tan H. (2019). Role of pyroptosis in cardiovascular diseases and its therapeutic implications. Int. J. Biol. Sci. 15, 1345–1357. 10.7150/ijbs.33568 31337966 PMC6643148

[B34] Zhang J. Liang Y. Huang X. Guo X. Liu Y. Zhong J. (2019). STAT3-induced upregulation of lncRNA MEG3 regulates the growth of cardiac hypertrophy through miR-361-5p/HDAC9 axis. Sci. Rep. 9, 460. 10.1038/s41598-018-36369-1 30679521 PMC6346020

[B35] Zhang L. Ai C. Bai M. Niu J. Zhang Z. (2022). NLRP3 inflammasome/pyroptosis: a key driving force in diabetic cardiomyopathy. Int. J. Mol. Sci. 23, 10632. 10.3390/ijms231810632 36142531 PMC9501057

[B36] Zhang Y. Liu X. Bai X. Lin Y. Li Z. Fu J. (2018). Melatonin prevents endothelial cell pyroptosis via regulation of long noncoding RNA MEG3/miR-223/NLRP3 axis. J. Pineal Res. 64, e12449. 10.1111/jpi.12449 29024030

[B37] Zhao X. Liu S. Wang X. Chen Y. Pang P. Yang Q. (2022). Diabetic cardiomyopathy: clinical phenotype and practice. Front. Endocrinol. (Lausanne) 13, 1032268. 10.3389/fendo.2022.1032268 36568097 PMC9767955

[B38] Zheng D. Liu J. Piao H. Zhu Z. Wei R. Liu K. (2022). ROS-triggered endothelial cell death mechanisms: focus on pyroptosis, parthanatos, and ferroptosis. Front. Immunol. 13, 1039241. 10.3389/fimmu.2022.1039241 36389728 PMC9663996

[B39] Zhu X. Li H. Wu Y. Zhou J. Yang G. Wang W. (2019). lncRNA MEG3 promotes hepatic insulin resistance by serving as a competing endogenous RNA of miR-214 to regulate ATF4 expression. Int. J. Mol. Med. 43, 345–357. 10.3892/ijmm.2018.3975 30431065 PMC6257836

[B40] Zou L. Ma X. Lin S. Wu B. Chen Y. Peng C. (2019). Long noncoding RNA-MEG3 contributes to myocardial ischemia–reperfusion injury through suppression of miR-7-5p expression. Biosci. Rep. 39, BSR20190210. 10.1042/bsr20190210 31366567 PMC6702358

