# Supplementary material for: LncRNA MEG3 exacerbates diabetic cardiomyopathy via activating pyroptosis signaling pathway
Source: Front Pharmacol. 2025 Apr 2;16:1538059. doi: 10.3389/fphar.2025.1538059 (PMC12000004; doi:10.3389/fphar.2025.1538059)
Supplement: Supplementary file 1 [file Table1.docx]

**Table S1. The primer sequences used in Real-Time polymerase chain reaction assays**

| Gene | Forward primer (5' to 3') | Reverse primer (5' to 3') |
| --- | --- | --- |
| lncMEG3 (mus) | AGGACTTCACGCACAACAC | CGATTTACAGTTGGAGGGTC |
| lncMEG3 (has) | TTGTCCACGGGCTCTCCTTG | CCACGGAGTAGAGCGAGTCA |
| miR-223 | GGGGTGTCAGTTTGTCAA | TATCCAGTGCGTGTCGTGGA |
| ANP (mus) | TACAGTGCGGTGTCCAACACAG | TGCTTCCTCAGTCTGCTCACTC |
| BNP (mus) | TCCTAGCCAGTCTCCAGAGCAA | GGTCCTTCAAGAGCTGTCTCTG |
| β-MHC | CCTGCGGAAGTCTGAGAAGG | CTCGGGACACGATCTTGGC |
| NLRP3 (mus) | GTGGAGATCCTAGGTTTCTCTG | CAGGATCTCATTCTCTTGGATC |
| ASC (mus) | GACAGTGCAACTGCGAGAAG | CGACTCCAGATAGTAGCTGACAA |
| Caspase-1 (mus) | ACACGTCTTGCCCTCATTATCT | ATAACCTTGGGCTTGTCTTTCA |
| IL-18 (mus) | GACAGCCTGTGTTCGAGGATATG | TGTTCTTACAGGAGAGGGTAGAC |
| IL-1β (mus) | TGGACCTTCCAGGATGAGGACA | GTTCATCTCGGAGCCTGTAGTG |
| NLRP3 (has) | GGACTGAAGCACCTGTTGTGCA | TCCTGAGTCTCCCAAGGCATTC |
| ASC (has) | CTGACGGATGAGCAGTACCA | CAGGATGATTTGGTGGGATT |
| Caspase-1 (has) | TTTCCGCAAGGTTCGATTTTCA | GGCATCTGCGCTCTACCATC |
| IL-18 (has) | TCTTCATTGACCAAGGAAATCGG | TCCGGGGTGCATTATCTCTAC |
| IL-1β (has) | ATGATGGCTTATTACAGTGGCAA | GTCGGAGATTCGTAGCTGGA |
| GAPDH | AAGAAGGTGGTGAAGCAGGC | TCCACCACCCTGTTGCTGTA |
