# Supplementary material for: LncRNA MEG3 exacerbates diabetic cardiomyopathy via activating pyroptosis signaling pathway
Source: Front Pharmacol. 2025 Apr 2;16:1538059. doi: 10.3389/fphar.2025.1538059 (PMC12000004; doi:10.3389/fphar.2025.1538059)
Supplement: Supplementary file 2 [file DataSheet1.pdf]

Figure 3 (1)

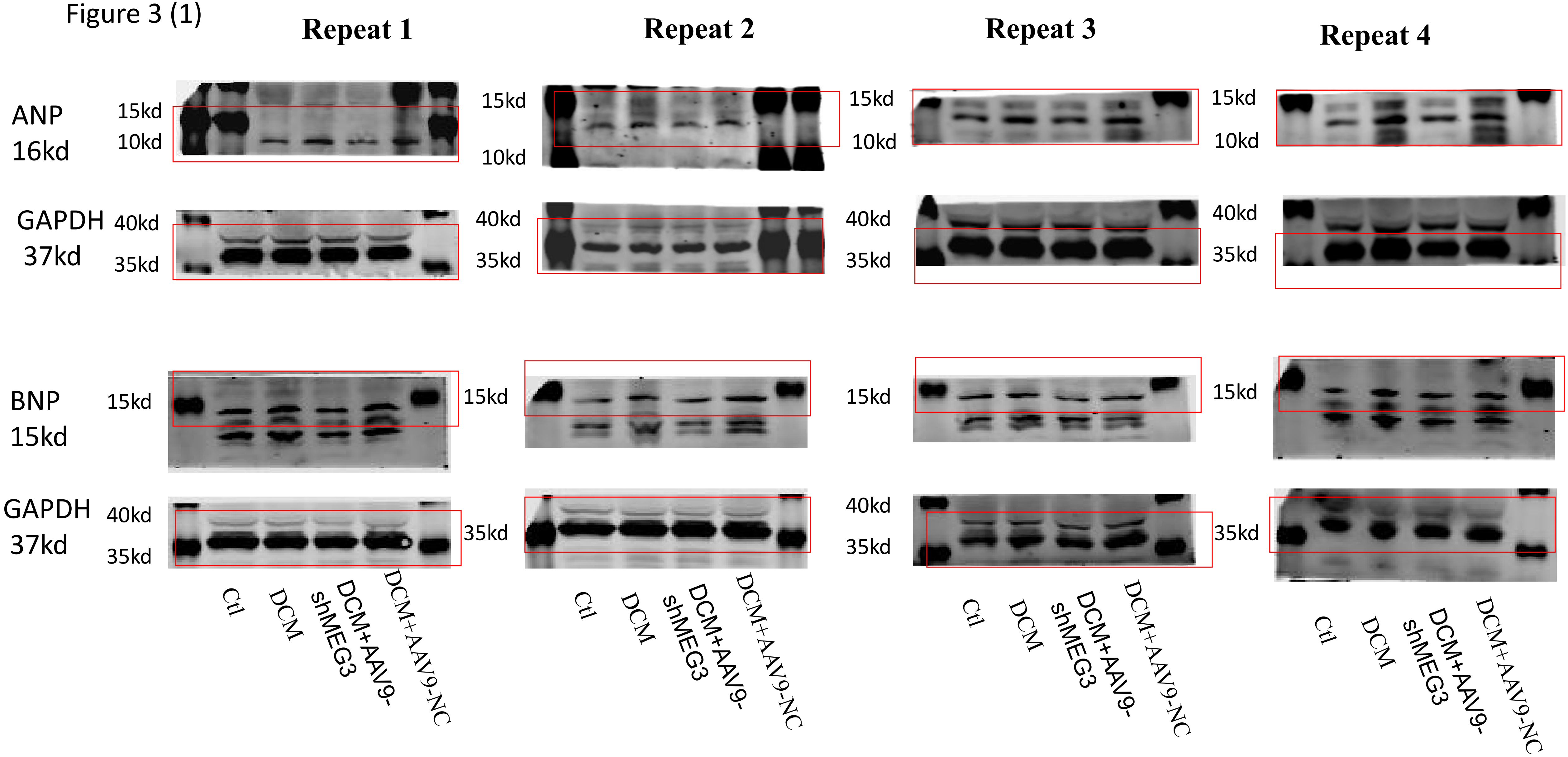
Repeat 1
Repeat 2
Repeat 3
Repeat 4
ANP
16kd
GAPDH
37kd
BNP
15kd
GAPDH
37kd
15kd
10kd
40kd
35kd
Ctl
DCM
DCM+AAV9-shMEG3
DCM+AAV9-NC

Figure 3 (2)

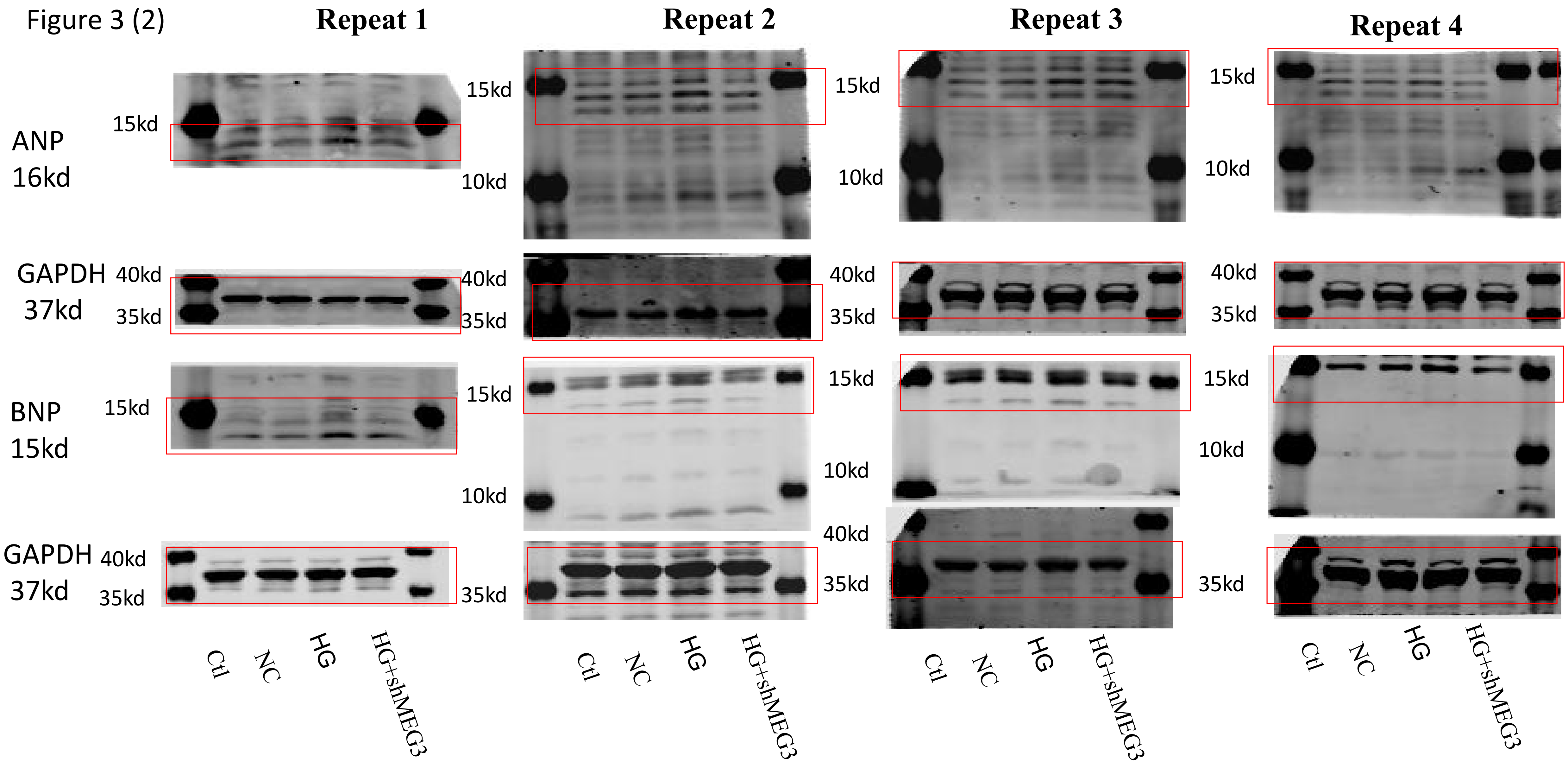
Repeat 1
Repeat 2
Repeat 3
Repeat 4
ANP
16kd
GAPDH
37kd
BNP
15kd
GAPDH
37kd
15kd
10kd
40kd
35kd
Ctl
NC
HG
HG+shMEG3

Figure 4 (1)

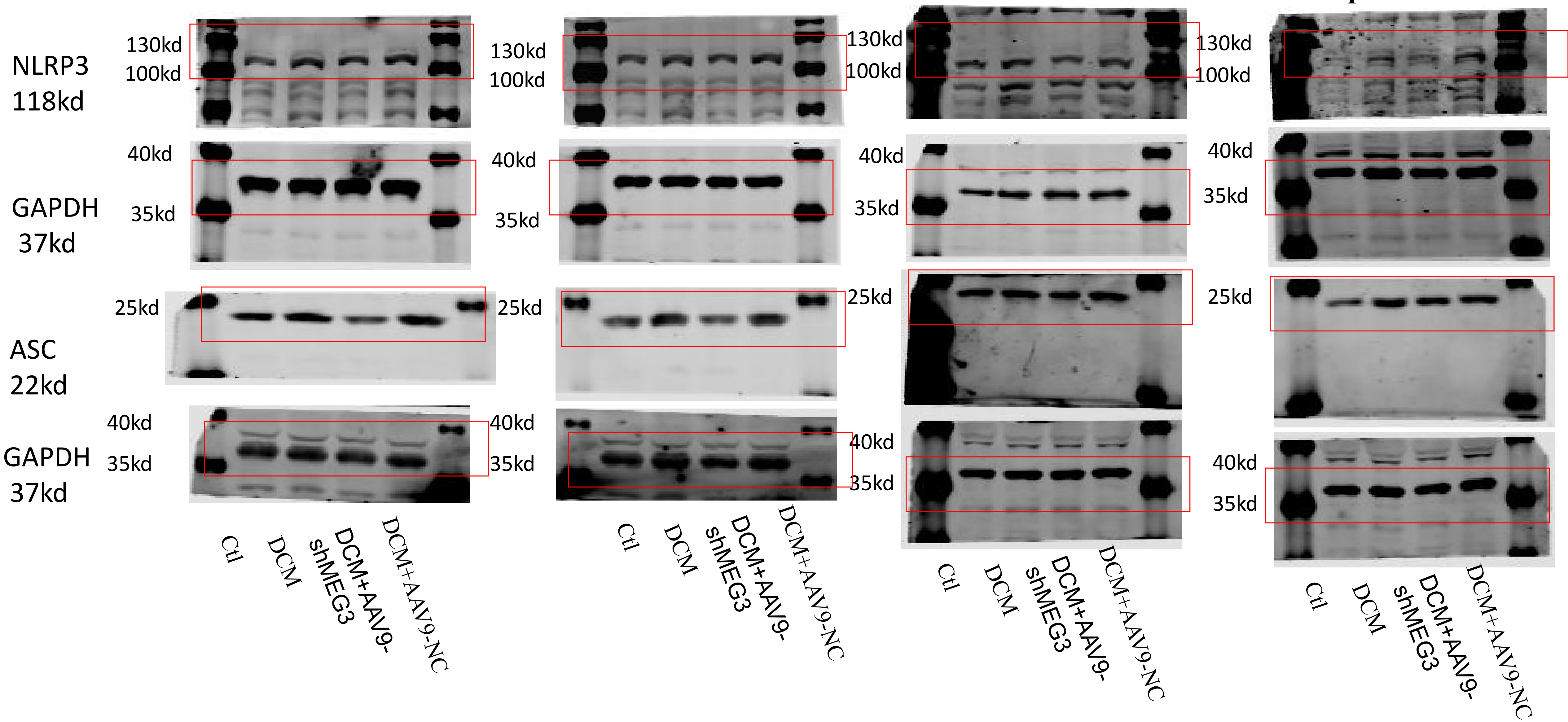
Repeat 1
Repeat 2
Repeat 3
Repeat 4
NLRP3
118kd
GAPDH
37kd
ASC
22kd
GAPDH
37kd
130kd
100kd
40kd
35kd
25kd
Ctl
DCM
DCM+AAV9-shMEG3
DCM+AAV9-NC

Figure 4 (2)

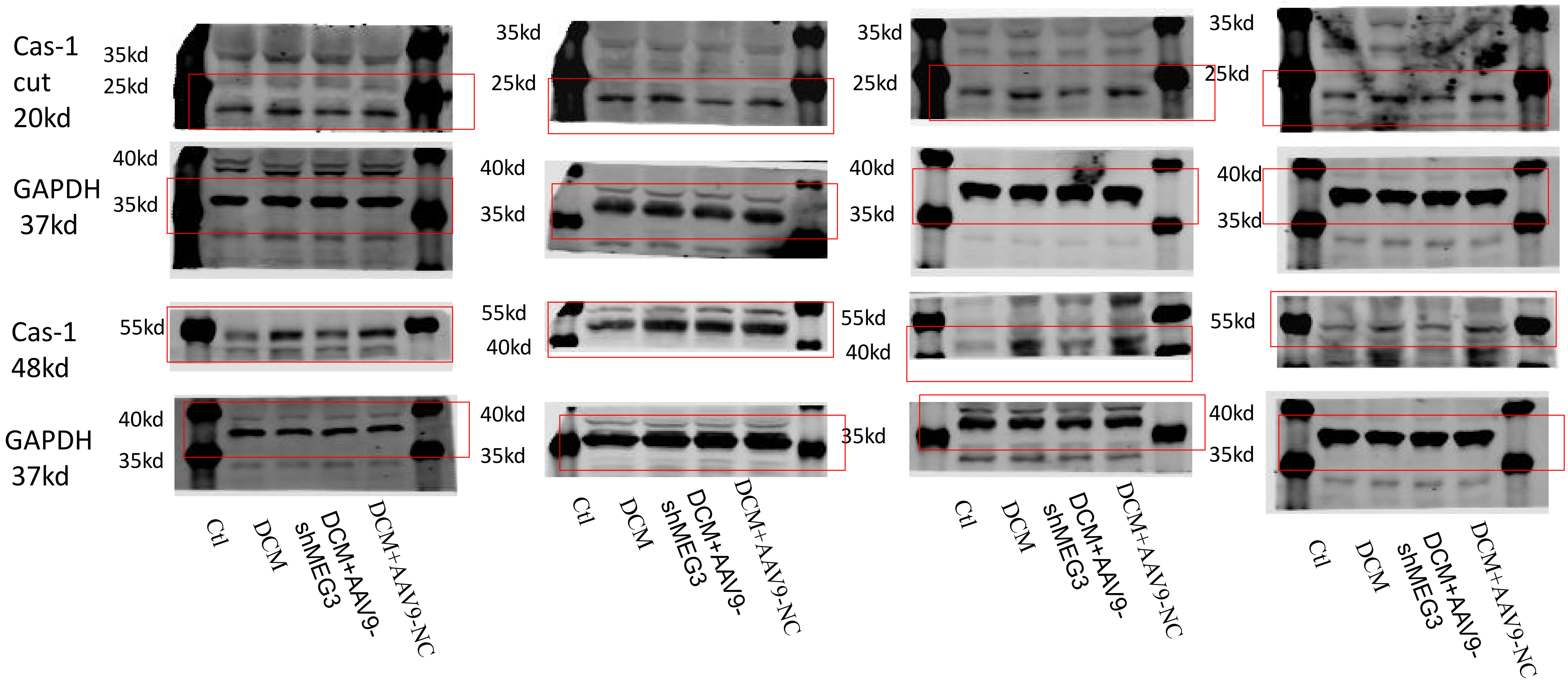
Repeat 1
Repeat 2
Repeat 3
Repeat 4
Cas-1
cut
20kd
GAPDH
37kd
Cas-1
48kd
GAPDH
37kd
35kd
25kd
40kd
35kd
55kd
40kd
Ctl
DCM
DCM+AAV9-
shMEG3
DCM+AAV9-NC

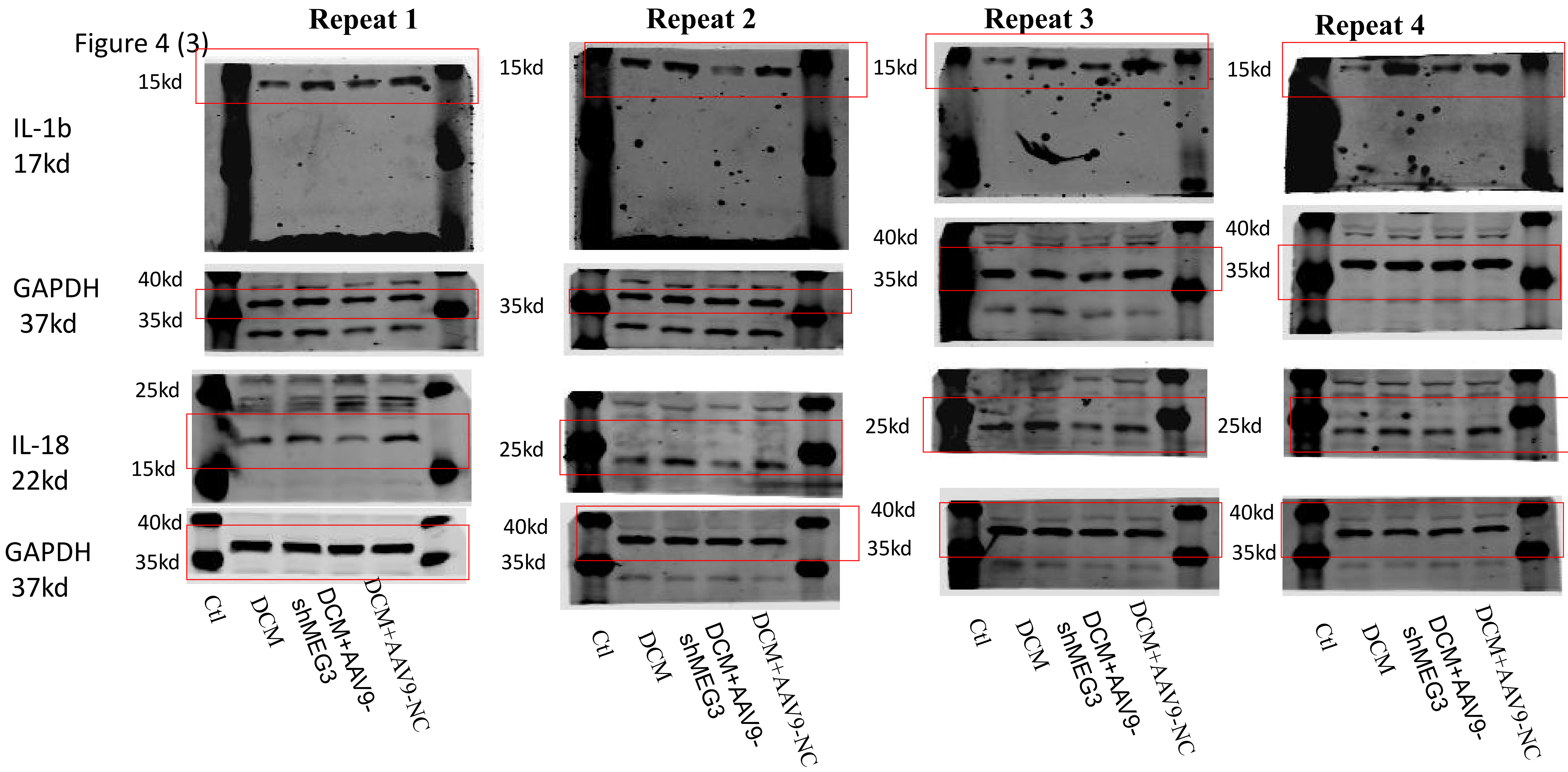

Figure 4 (3)
Repeat 1
Repeat 2
Repeat 3
Repeat 4
IL-1b
17kd
GAPDH
37kd
IL-18
22kd
GAPDH
37kd
15kd
40kd
35kd
25kd
Ctl
DCM
DCM+AAV9-shMEG3
DCM+AAV9-NC

Figure 5 (1)

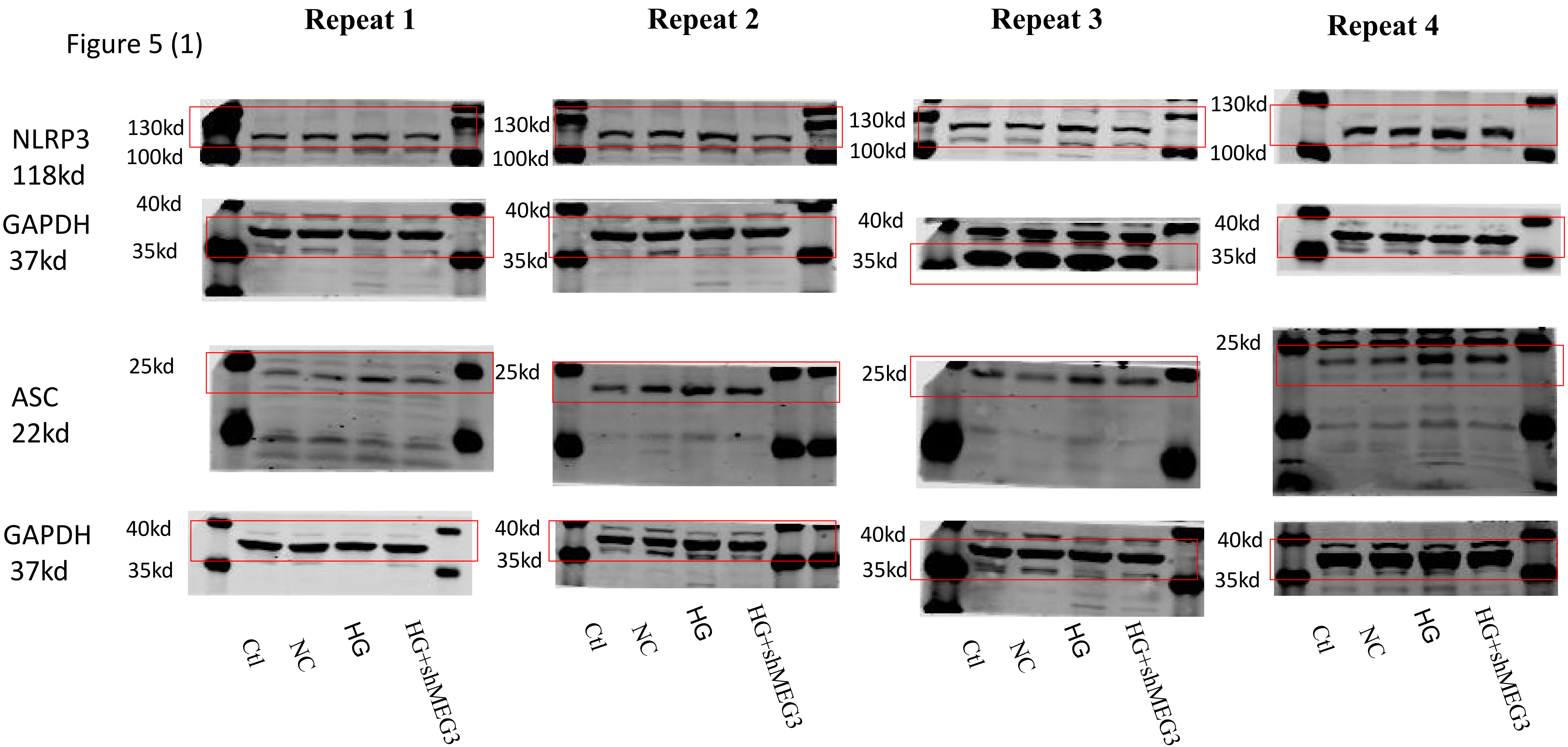

Repeat 1
Repeat 2
Repeat 3
Repeat 4
NLRP3
118kd
GAPDH
37kd
ASC
22kd
GAPDH
37kd
130kd
100kd
40kd
35kd
25kd
Ctl
NC
HG
HG+shMEG3

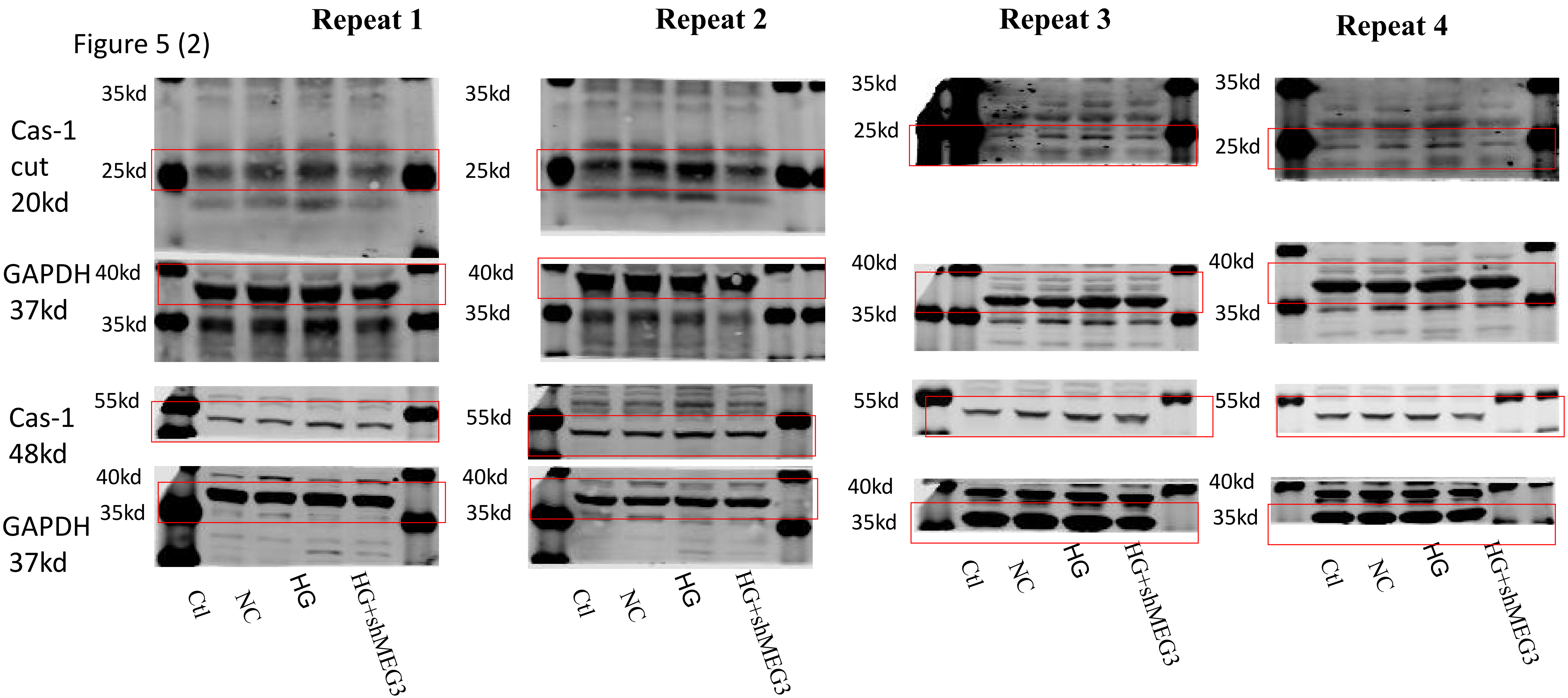

Figure 5 (2)
Repeat 1
Repeat 2
Repeat 3
Repeat 4
Cas-1
cut
20kd
GAPDH
37kd
Cas-1
48kd
GAPDH
37kd
35kd
25kd
40kd
55kd
Ctl
NC
HG
HG+shMEG3

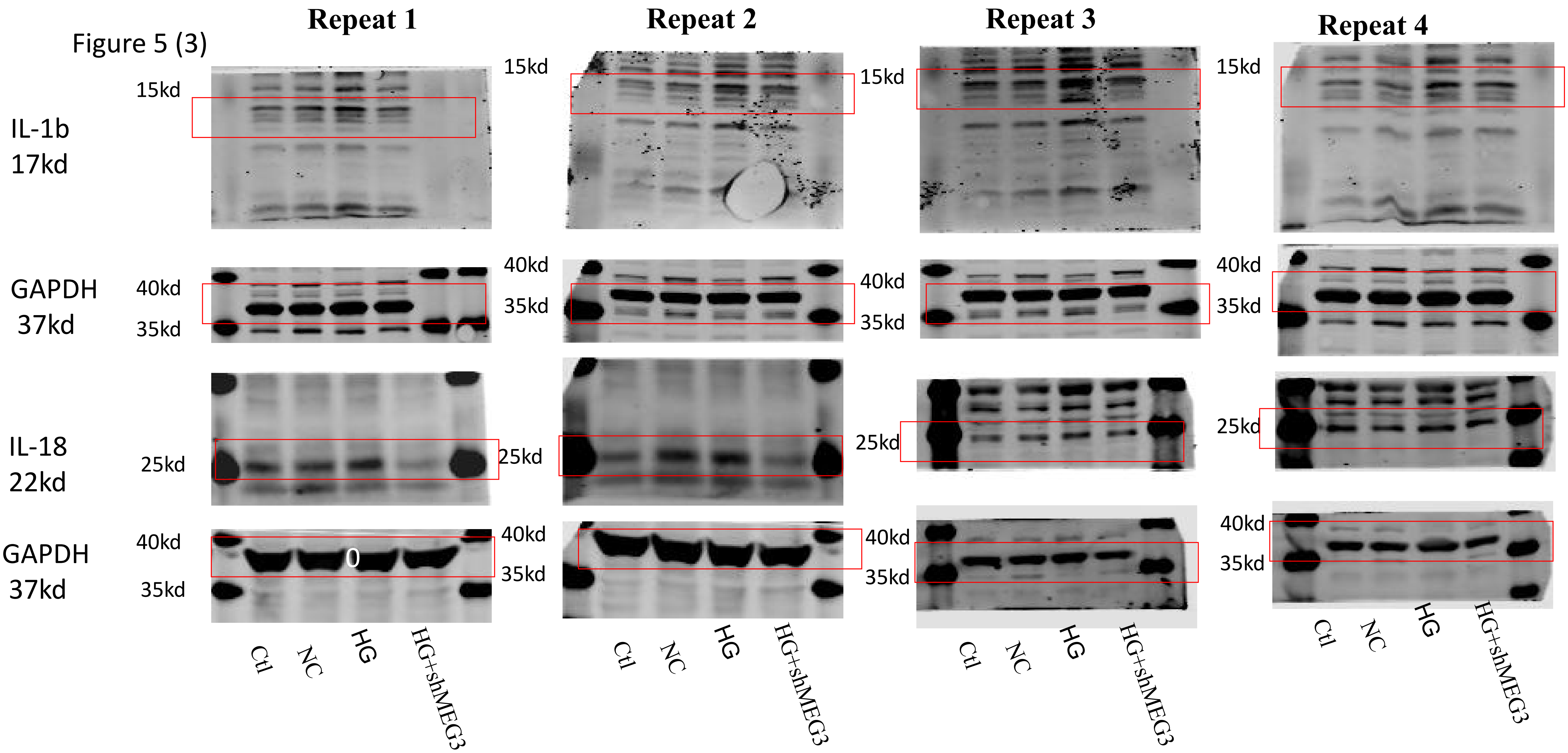

Figure 5 (3)
Repeat 1
Repeat 2
Repeat 3
Repeat 4
IL-1b
17kd
GAPDH
37kd
IL-18
22kd
GAPDH
37kd
15kd
40kd
35kd
25kd
Ctl
NC
HG
HG+shMEG3

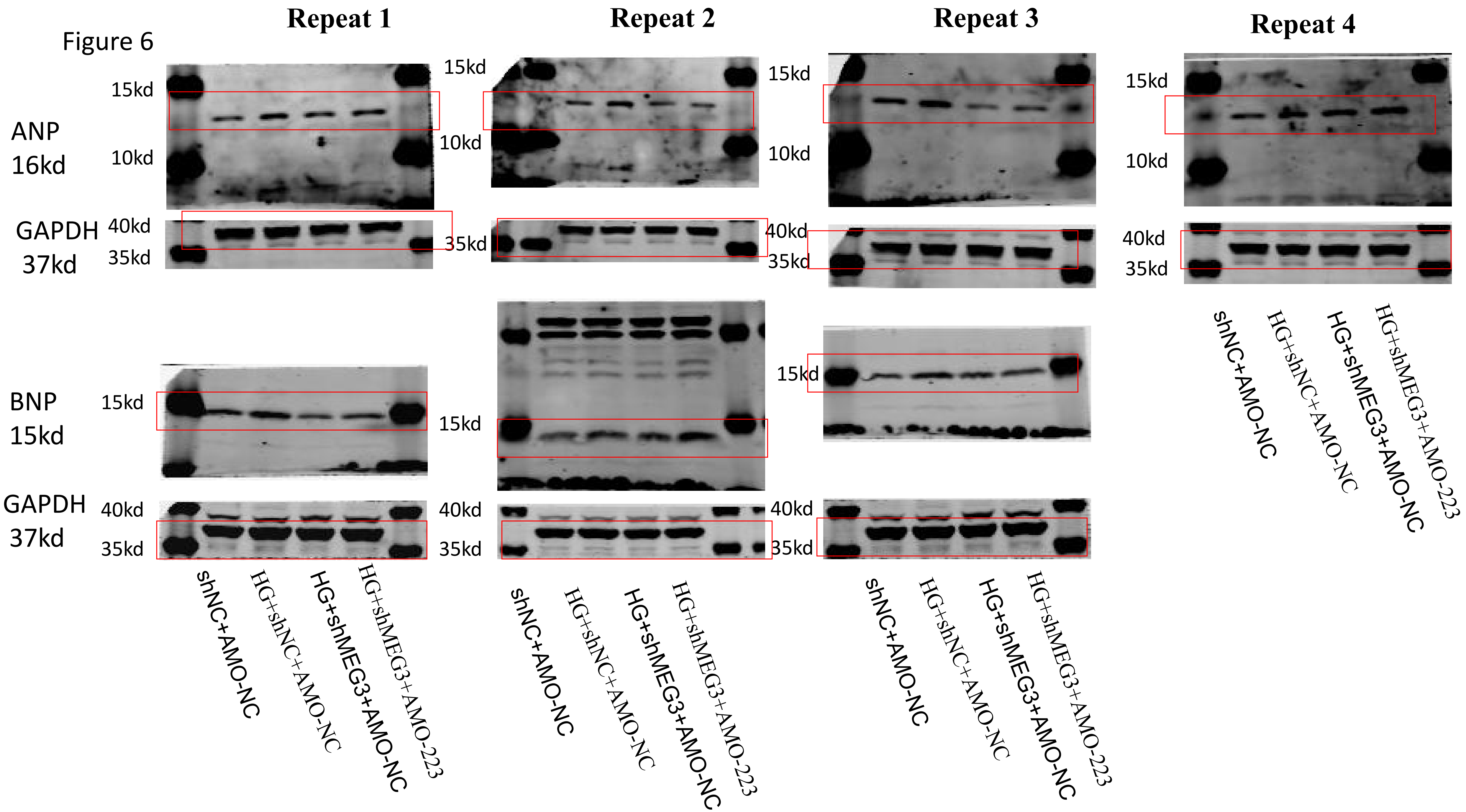
Figure 6
Repeat 1
Repeat 2
Repeat 3
Repeat 4
ANP
16kd
GAPDH
37kd
BNP
15kd
GAPDH
37kd
15kd
10kd
40kd
35kd
shNC+AMO-NC
HG+shNC+AMO-NC
HG+shMEG3+AMO-NC
HG+shMEG3+AMO-223

Figure 7 (1)

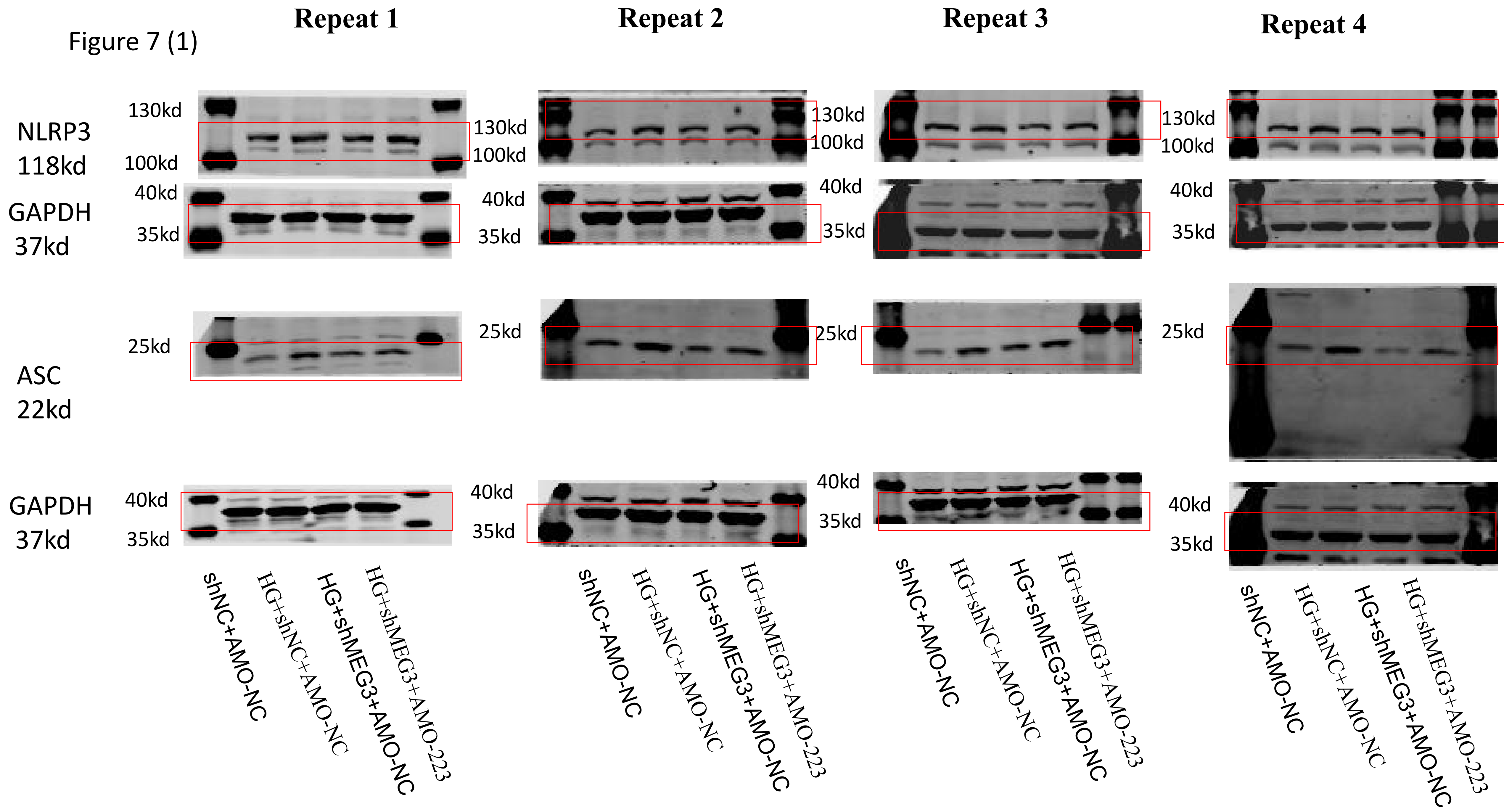
Repeat 1
Repeat 2
Repeat 3
Repeat 4
NLRP3
118kd
GAPDH
37kd
ASC
22kd
GAPDH
37kd
130kd
100kd
40kd
35kd
25kd
shNC+AMO-NC
HG+shNC+AMO-NC
HG+shMEG3+AMO-NC
HG+shMEG3+AMO-223

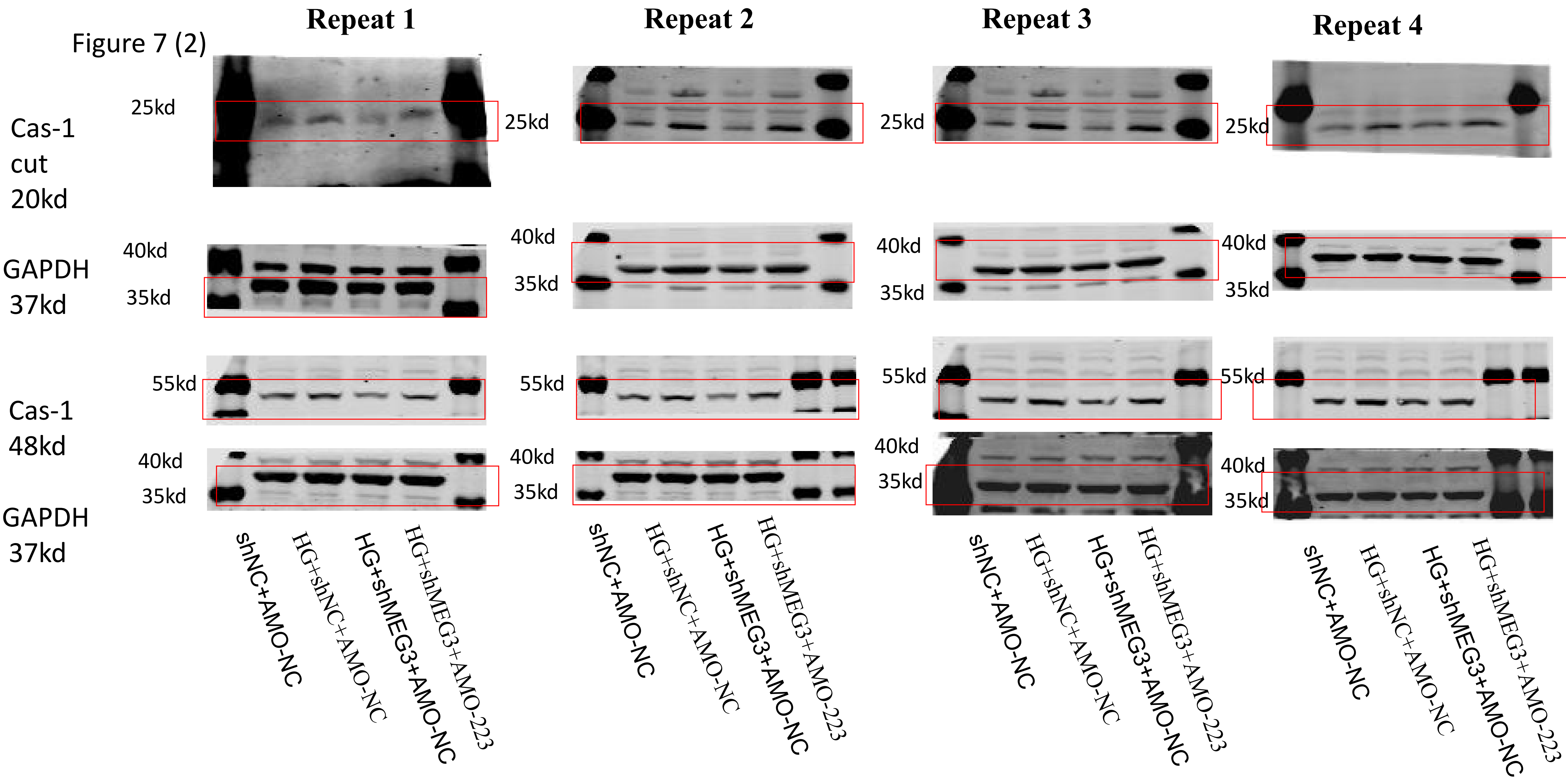
Figure 7 (2)
Repeat 1
Repeat 2
Repeat 3
Repeat 4
Cas-1 cut 20kd
GAPDH 37kd
Cas-1 48kd
GAPDH 37kd
25kd
40kd
35kd
55kd
shNC+AMO-NC
HG+shNC+AMO-NC
HG+shMEG3+AMO-NC
HG+shMEG3+AMO-223

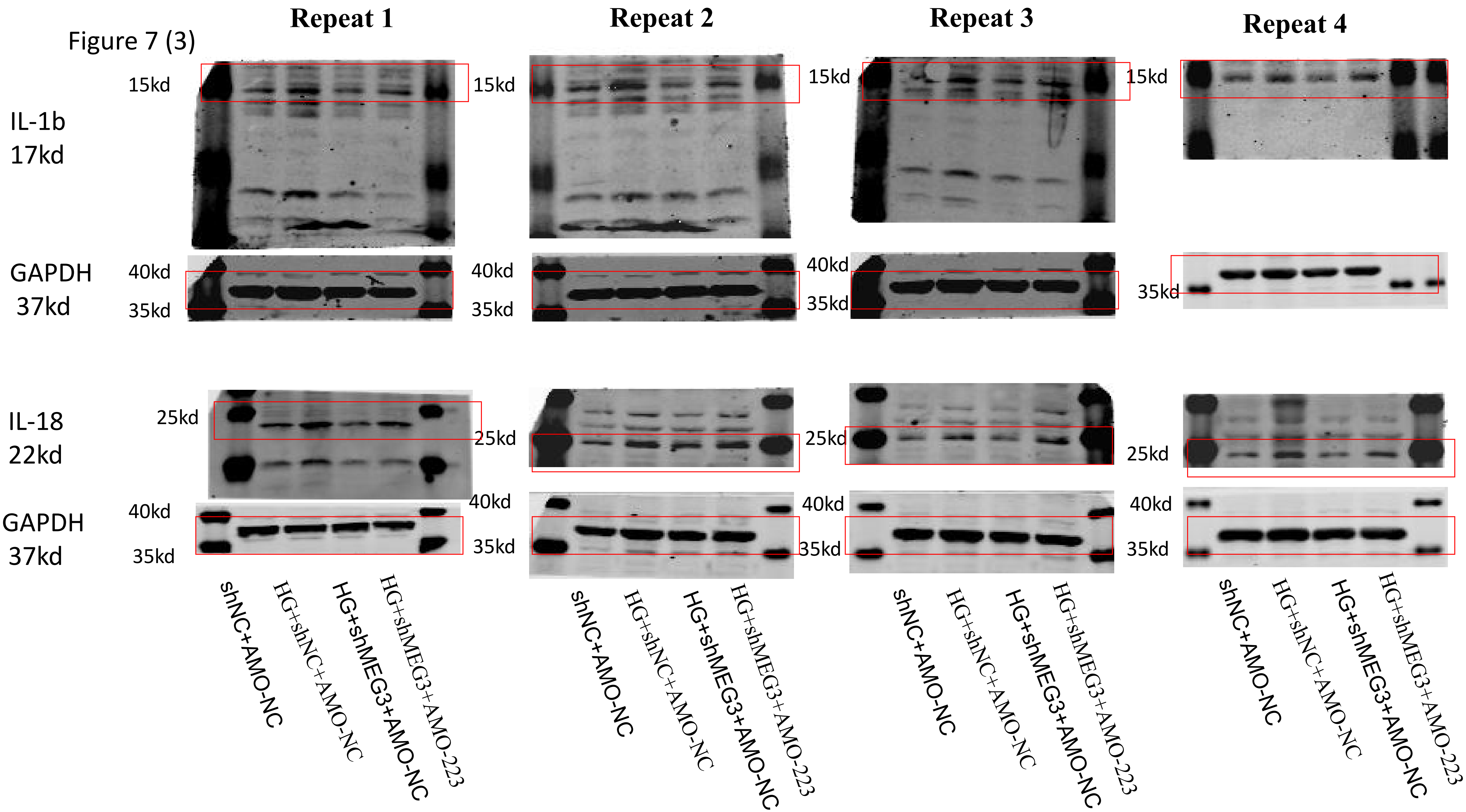

Figure 7 (3)
Repeat 1
Repeat 2
Repeat 3
Repeat 4
IL-1b
17kd
15kd
GAPDH
37kd
40kd
35kd
IL-18
22kd
25kd
shNC+AMO-NC
HG+shNC+AMO-NC
HG+shMEG3+AMO-NC
HG+shMEG3+AMO-223
